# Case report: Endolymphatic system disease in elasmobranchs: clinical presentation, diagnosis, and treatment strategies

**DOI:** 10.3389/fvets.2024.1463428

**Published:** 2025-03-10

**Authors:** Whitney Greene, Nuno Pereira, Bethany Doescher, Carlos Rojo-Solis, Hugo David, Ricardo Faustino, David Reese, Ryan De Voe, Ed Latson, Natalie Mylniczenko

**Affiliations:** ^1^Mote Marine Laboratory and Aquarium, Sarasota, FL, United States; ^2^Oceanário de Lisboa, Esplanada D. Carlos I, Lisbon, Portugal; ^3^Sea Life Park Hawaii, Waimanalo, HI, United States; ^4^Oceanogràfic, Ciudad de las Artes y las Ciencias, Valencia, Spain; ^5^Research Unit in Medical Imaging and Radiotherapy, Cross I&D Lisbon Research Center, Escola Superior de Saúde da Cruz Vermelha Portuguesa, Lisbon, Portugal; ^6^VetCT Consultants in Telemedicine PTY LTD., Applecross, WA, Australia; ^7^Disney Animals, Science and Environment, Disney’s Animal Kingdom^®^ and the Seas with Nemo and Friends^®^, Lake Buena Vista, FL, United States; ^8^Aquarium of Niagara, Niagara Falls, NY, United States; ^9^Ripley’s Marine Science Center, Cheektowaga, NY, United States

**Keywords:** shark, endolymphatic pore, labyrinthitis, inner ear, otic disease, neurologic disease, neuropathy, stingray

## Abstract

The inner ear is an often overlooked system in elasmobranchs with few documented reports of disease or other abnormalities in the literature. Similar to terrestrial vertebrates, it is located in the cranium, and there are multiple components to the ear of elasmobranchs including a pair of membranous labyrinths each with three semicircular canals and four chambers or end organs (the saccule, the lagena, the utricle and the macula neglecta) making up the endolymphatic system (ELS). There is species variability among the inner ear anatomy of elasmobranchs, and this may play a role in disease development, progression, and treatment outcomes. Also similar to terrestrial vertebrates, this system plays a key role in hearing, acceleration, and orientation. When affected, clinical signs may include localized areas of swelling or stoma development along the dorsal midline of the head at the endolymphatic pores, atypical swimming behaviors consistent with vestibular disease (spiraling/spinning or barrel rolling, or tilting to one side), and anorexia. Less frequently, the eyes may also be affected and present with exophthalmia, hyphema, and/or panophthalmitis. Herein are case series from five institutions representing a variety of elasmobranch species affected with ELS disease with discussion of anatomy, clinical presentation, diagnostics, etiology, treatment, and outcomes. Endolymphatic disease may be clinically underdiagnosed in elasmobranchs and mistaken for other diseases such as superficial subcutaneous or subdermal abscesses, focal dermatitis, or neuropathies presumed to not be associated with the inner ear system. In addition, disease may be occult for a long period of time prior to overt manifestation of signs or chronic with waxing and waning clinical signs, likely because of anatomy and resultant treatment challenges. Awareness and additional research may help to promote timely identification, improve diagnostic and treatment options, and help to optimize individual animal welfare.

## Introduction

The inner ear is an often overlooked system in elasmobranch medicine, and there are few documented reports of disease in the literature (1–4). Clinical signs are highly varied and may include abnormal swimming patterns such as barrel rolling, spiraling, spinning, tilting to one side, nodules/abscesses in the dorsal surface of the head, anorexia, and associated ophthalmic disease such as hyphema, conjunctivitis, exophthalmia, and panophthalmitis (1, 4).

The ear of elasmobranchs is located within the caudal chondrocranium (5, 6). There are bilateral membranous labyrinths each containing three semicircular canals and four sensory maculae (7–14). Elasmobranchs only possess inner ear labyrinths and have no other accessory organs of hearing (6). There are differences between elasmobranch species with inner ear variation being characterized by three primary axes (15, 16) that are influenced by diet and habitat. Piscivorous elasmobranchs have larger inner ears compared to non-piscivorous species, and reef-associated species have larger inner ears compared to oceanic species (17). Despite a similar inner ear blueprint that is consistent among most fishes, there is considerable variation between species in the size and shape of various structures (17) with the relevant difference between terrestrials and elasmobranchs being an enlarged macula neglecta (8, 12, 18).

**Figure 1:**
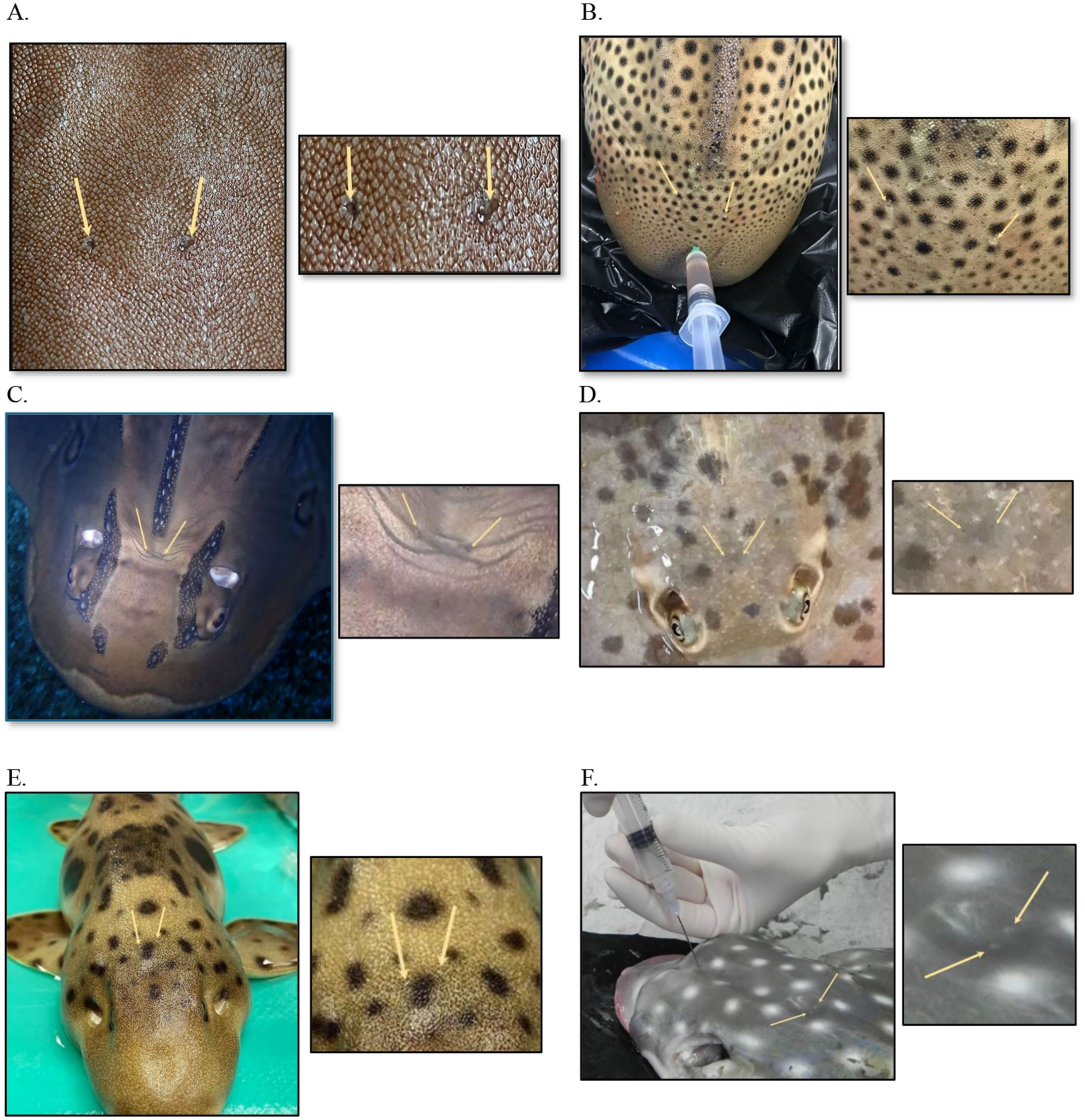
Endolymphatic pores in normal elasmobranchs. **(A)** Bamboo shark (*Chiloscyllium punctatum*), close up. **(B)** Zebra shark (*Stegostoma tigrinum*) post-mortem cerebrospinal fluid collection. **(C)** Bowmouth guitarfish (*Rhina ancylostoma*). **(D)** Chilean ray (*Urotrygon chilensis*). **(E)** Coral catshark (*Atelomycterus marmoratus*). **(F)** Spotted eagle ray (*Aetobatus narinari*) cerebrospinal fluid collection, post-mortem.

**Figure 2:**
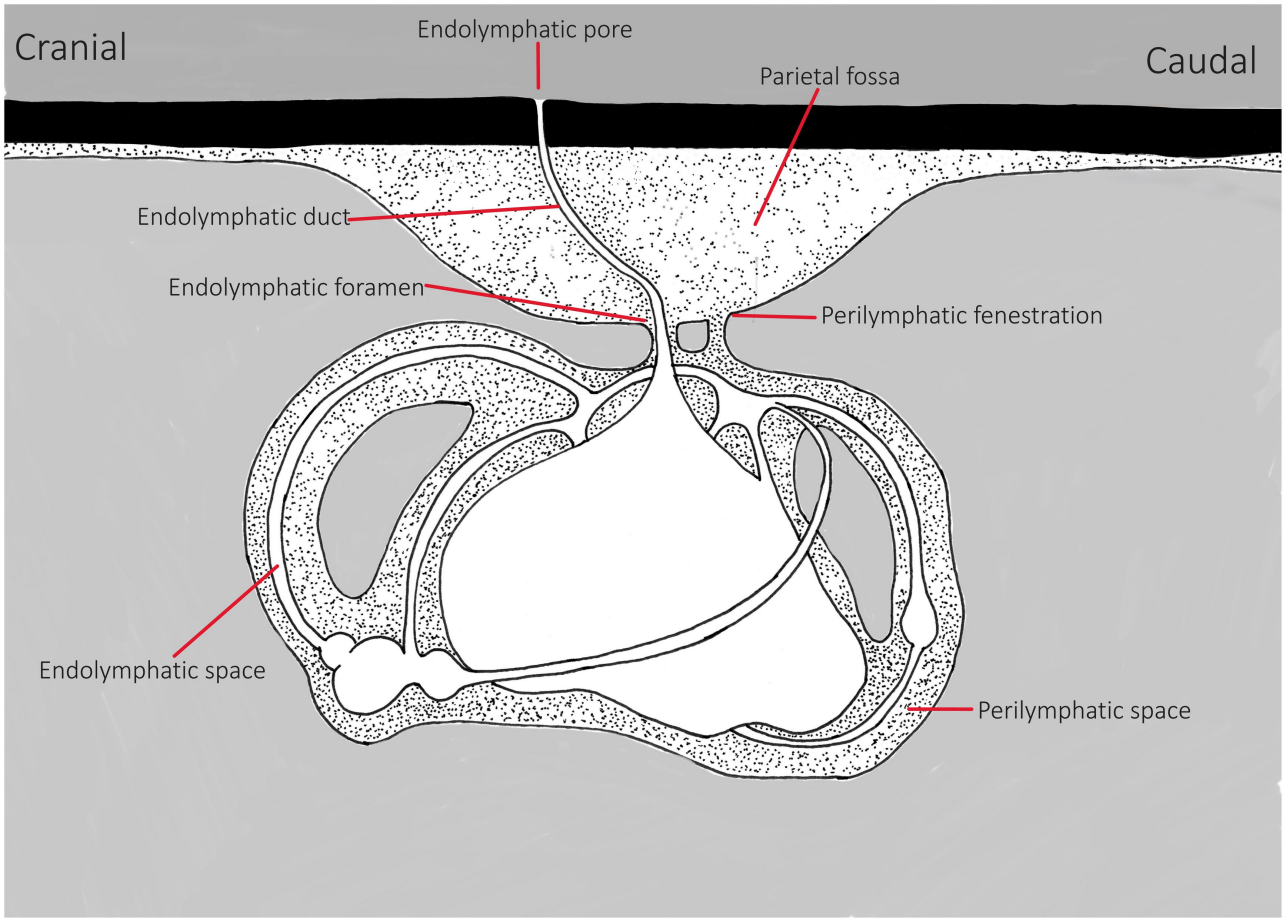
Line drawing of basic inner ear of elasmobranchs, representing general anatomy. This is a representation, differences may include variations in size/depth of the parietal fossa, in the endolymphatic duct’s direction and width/volume and shape of the perilymph bathing the labyrinth within the chondrocranium and the size of the end organs. Adapted from Chang and Okihiro (5).

The only external indicator of the position of the ears is the presence of small, paired endolymphatic pores (Figure 1) found in the skin on the dorsal chondrocranium. They may or may not be easily visible in healthy animals (5, 13, 19). The endolymphatic pores lie adjacent to one another close to the medial line, just anterior to the supratemporal canal of the lateral line (14), and they connect the inner ear to the outside environment (20) (Figure 2). Each pore leads to the inner ear via paired endolymphatic ducts (or sacs) through the parietal fossa into the endolymphatic foramen within the chondrocranium. The ducts then lead to the otic organs and are lined with small, paired muscles but overall are thin and fragile (5, 11). The fossa can be variably sized with some being spacious, such as the Triakidae (16, 21).

Within the dorsal chondrocranium, there are two perilymphatic foramina (larger ovoid holes, posteromedial to the endolymphatic ducts) that are covered by a membrane (11, 22). These lead into bilateral perilymphatic filled spaces that house the labyrinth in the otic capsule (Figure 2) (5, 11, 13, 16, 21, 23). For a clinically relevant description, Corwin (16) suggests generalized classification of the ears into two categories: “A small ventrally located sacculus, a small ventrally located posterior canal duct, a small macula neglecta, and a small lateral perilymphatic fluid space or falling into the second category, with a large triangular sacculus, a large lateral fluid space, and a large, dorsally positioned posterior canal duct containing a well-developed macula neglecta.” These distinctions will have bearing on diagnosis and consideration for treatment strategies.

The endolymphatic ducts initially track rostrally and then retroflex in most species, extending ventrally and caudally in the chondrocranium (14, 23). The ducts are thin tubes that connect to the saccule. The endolymphatic ducts in some species may expand into a large sac into the fossa (11). The function of the ducts is unknown but believed to help with fluid exchange between the ear and the environment and may also function in wave displacement or for pressure equalization (14). Each vestibular chamber has an otolithic mass composed of small mineral crystals called otoconia, compacted by an organic matrix (22–24, 73). These otolithic masses are different from the solid and dense otoliths found in teleosts (13, 24, 25, 73). In some elasmobranchs, the otoconia consist of small particles of exogenous environmental sand (11, 25). There is more detailed literature on the anatomy and function of the endolymphatic system (ELS) and hearing in elasmobranchs; for brevity in this document, the readers are encouraged to further their review with these sources (5–7, 10–14, 17, 18, 20, 22–28).

In an effort to supplement the scarce literature describing clinical disease, the following series will describe the clinical signs, diagnosis, treatments, and outcomes of 23 cases of ELS disease in elasmobranchs (Table 1). These cases will be parsed into six separate case series based on the institution that oversaw the treatment and care of the individual animals. For this entire series, cases are defined by their involvement of the endolymphatic pores and surrounding structures or by direct evidence of involvement of other endolymphatic structures by imaging or pathology. Due to the complex nature of the disease and chronicity of many of these cases, salient points from their histories are listed in an effort to consolidate and discuss all of them. This disease process is likely underdiagnosed; it is the authors’ goal to discuss the etiology, clinical signs, diagnostic, and treatment options to help with case identification and management.

**Table 1:** Summary table of all endolymphatic system disease cases in twenty three elasmobranchs.

Case series #	Number	Species	Gender	Age	Superficial disease only	Complex disease (neurologic)	Recurrence of clinical signs	Occurrence	Culture	Died	Euthanasia	ELD Pathology (Gross exam or imaging)	Resolution	Comments
1	1	*Rhina ancylostomus*	Male	3	N	Y	N	0	*Enterococcus faecalis*	Y	Y	Y	N	*Enterococcus faecalis* cultured from blood and aspirate samples
	2	*Rhina ancylostomus*	Male	6	N	Y	Y	1	*Enterococcus faecalis*	Y	N	Y	N	Superficial on first presentation
	3	*Urobatis jamaicensis*	Male	8	N	Y	Y	1	*Mycobacterium chelonae*	Y	N	Y	N	Superficial on first presentation; *Mycobacterium chelonae* serially cultured
	4	*Urogymnus asperrimus*	Female	6	N	Y	Y	1	Negative	N	N	Y (imaging)	Y	Superficial on first presentation
	5	*Chiloscyllium plagiosum*	Female	9	Y	N	N	0	*Enterococcus faecalis*	N	N	Y (imaging)	Y	*Enterococcus faecalis* cultured from blood and aspirate samples
2	6	*Heterodontus portusjacksoni*	Female	~4	N	Y	N	0	*Fusarium solani*	Y	N	Y	N	Died quickly
	7	*Heterodontus portusjacksoni*	Female	~6	N	Y	N	0	*Fusarium solani*	Y	N	Y	N	
	8	*Heterodontus portusjacksoni*	Female	~6	N	Y	Y	1	*Fusarium solani*	Y	N	Y	N	Superficial on first presentation
	9	*Heterodontus portusjacksoni*	Male	~6	N	Y	N	0	*Fusarium solani*	Y	N	Y	N	
	10	*Heterodontus portusjacksoni*	Male	2	N	Y	N	0	*Fusarium solani, Citrobacter braaki, Stenotrophomonas maltophilia*	Y	N	Y	N	*Fusarium solani* (abscess and brain); *Citrobacter braaki* (abscess); S*tenotrophomonas maltophilia* (brain)
	11	*Heterodontus portusjacksoni*	Male	3	N	Y	N	0	*Fusarium solani*	Y	N	Y (imaging)	N	
3	12	*Mobula hypostoma*	Male	8	N	Y	Y	1	N/A	Y	Y	Y	N	
4	13	*Carcharias taurus*	Male	25	N	Y	Y	1	Negative	Y	N	Y	N	
	14	*Hypanus americanus*	Female	15	Y	N	N	0	N/A	N	N	N	Y	
5	15	*Carcharhinus plumbeus*	Female	7	Y	N	N	0	*Enterococcus faecalis*	Y	N	N	N	Meningitis and mild perivascular lymphoplasmacytic encephalitis
	16	*Carcharhinus plumbeus*	Female	7	Y	N	Y	1	*Enterococcus faecalis*	Y	Y	N	N	
	17	*Chiloscyllium plagiosum*	Female	4	N	Y	Y	1	*Enterococcus faecalis*	Y	Y	N	N	Signs resolved but recurred after transport to new facility
6	18	*Triaenodon obesus*	Female	3	N	Y	Y	1	*Enterococcus faecalis*	Y	N	N	Y	Resolved before death
	19	*Triaenodon obesus*	Female	4	N	Y	Y	7	*Enterococcus faecalis*	Y	Y	N	Y	Recurred seven times in three years; Resolved before death
	20	*Triaenodon obesus*	Male	2	Y	N	N	0	*Enterococcus faecalis*	Y	N	Y	N	Initially no neurologic signs, asymptomatic 5 yrs., then symptomatic. Cancer.
	21	*Triaenodon obesus*	Female	2	Y	N	Y	2	*Enterococcus faecalis; Staphylococcus epidermidis-*methicillin resistant	Y	N	N	Y	Resolved before death
	22	*Triaenodon obesus*	Female	1	Y	N	Y	5	*Enterococcus faecalis*	Y	N	N	Y	Resolved before death
	23	*Triaenodon obesus*	Female	<1	Y	N	Y	10	*Enterococcus faecalis*	N	N	N	N	Continues to recur

### Case series 1

#### Case 1: Bowmouth guitarfish

**Figure 3:**
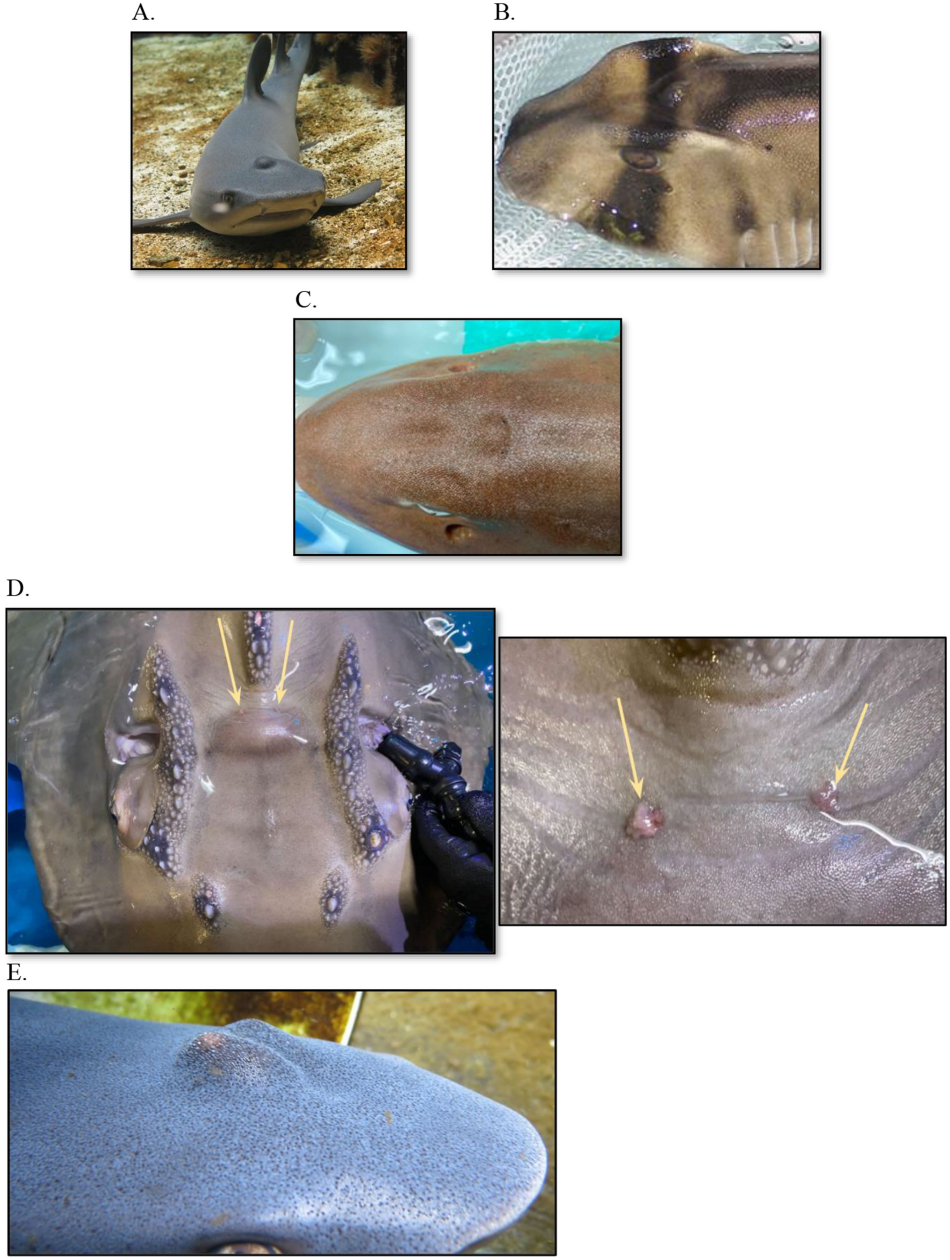
Swelling along the dorsal midline associated with endolymphatic system disease in various elasmobranch species. **(A)** White tip reef shark (*Triaenodon obesus*); **(B)** Port Jackson shark (*Heterodontus portusjacksoni*); **(C)** Bamboo shark (*Chiloscyllium punctatum*); **(D)** Bowmouth guitarfish (*Rhina ancylostoma*), zoomed out and up close; **(E)** White tip reef shark (*Triaenodon obesus*).

A 3-year-old male bowmouth guitarfish (*Rhina ancylostoma*) presented acutely for abnormal swimming consisting of barrel rolling and tilting to his left side. The differentials included scuticociliosis, central nervous system (CNS) disease (meningitis/encephalitis), and ELS disease. Physical examination revealed swollen endolymphatic pores (Figure 3) with purulent debris expelled on palpation. Initial workup consisted: of debridement, culture, cytology, blood work, cerebrospinal fluid (CSF) collection, and wound treatment. Serial blood work was performed throughout treatment and was overall unremarkable although *Enterococcus faecalis* was cultured from blood and pore samples. Empirical treatment was started with metronidazole (500 mg IV and PO SID; Pfizer, New York, NY, USA), doxycycline (2.5 mg/kg PO SID; Pfizer, New York, NY, USA), fluid support (250 mL elasmobranch ringers IV SID), oxytetracycline (4.1 mg/kg IM SID; Pfizer, New York, NY, USA), ketoprofen (6 mg/kg IV SID; Ketofen^®^, Zoetis, Parsippany-Troy Hills, NJ, USA), and meclizine (0.5 mg/kg PO SID; Trigen Laboratories, 2,631 Causeway Center Dr., Tampa, FL, USA). The guitarfish initially improved clinically (increased appetite and decrease in clinical signs) on this treatment but then began to subsequently decline. Secondary wounds developed along the ventral surface and fins, and additional neurologic signs presented (increased spinning frequency and severity). Metronidazole drug levels were found to be high at 96 μg/mL (29, 30) on day 6 of treatment coincident with these increased neurologic signs (Table 2). Metronidazole was discontinued, misoprostol and phenytoin powder (LS Polyox puffer 5 g per gram; Wedgewood Pharmacy, Swedesboro, NJ, USA) was initiated topically, and the guitarfish was transferred to a larger holding area with clinical improvement once metronidazole was stopped.

**Table 2:** Drug level comparison between different species.

Drug	Species	Drug level/C_max_	Units	Dose (mg/kg)	Source
Carprofen	Bowmouth Guitarfish 2	0.7; 0.6; 0.303	μg/mL	5	Case series 1
	Rainbow trout	2.52	μg/mL	2.5	(62)
	Canine (domestic)	5.43–35.30	μg/mL	0.7–4.0	(63)
	Quail	6.53	μg/mL	10	(64)
Meclizine	Yellow Ray	1,500; 2000; 2,900	μg/mL	1.5	Case series 1
	Bowmouth Guitarfish 2	140	μg/mL	0.5	Case series 1
Metronidazole	Bowmouth Guitarfish 1	61; 40; 65	μg/mL	6	Case series 1
	Bowmouth Guitarfish 2	170	μg/mL	30	Case series 1
	Canine (domestic)	8.84 ± 5.4	μg/mL	5 (IV); 20 (PO)	(65)
	Geese	60.27	μg/mL	50	(66)
	Horses	22+/−8 (PO); 9+/−2	μg/mL	20	(67)
Ketoprofen	Bowmouth Guitarfish 1	0; 0; 0; 0	mcg/ml	4; 5; 6	Case series 1
	Canine (domestic)	2.02 ± 0.41 (PO); 0.54	μg/mL	1 (PO); 10 (TDK)	(68)
	Nile Tilapia	18.15 (IM); 24.07 (IV)	μg/mL	3 (IM & IV)	(69)
Tramadol	Bowmouth Guitarfish 1	170	μg/mL	4.8	Case series 1
	Canine (domestic)	2.52 ± 0.43	μg/mL	4 (IM)	(70)
	Muscovy Ducks	0.78	μg/mL	30	(71)

Due to progression of the skin wounds, the patient was transferred to a large isolated animal holding area where he declined further. The guitarfish became completely anorectic and began displaying increasing periods of resting behavior and continued disorientation. The wounds continued to progress, and blood work revealed increasing and progressive inflammation and anemia. Parasites and CNS disease were ruled out through diagnostics, with ELS disease identified as the primary differential. Treatments were increased to include daily assist feeding (~1,150 mL gruel SID), ampicillin (18 mg/kg IV SID; Pfizer, New York, NY, United States), polymyxin B (15,000 IU/kg (1,250,000 IU) IV SID; Xellia Pharmaceuticals, Copenhagen, Denmark), fluid support (elasmobranch ringers 250 mL IV SID), prednisone (0.25–0.5 mg/kg PO SID; Jubilant Cadista Pharmaceuticals Inc., 207 Kiley Dr., Salisbury, MD, USA), tramadol (3.6 mg/kg PO SID; ULTRAM^®^, Cipher Pharmaceuticals, 5,750 Explorer Dr., Mississauga, ON L4W 5 K9, Canada), gabapentin (3.7 mg/kg PO SID; Pfizer, New York, NY, USA), meclizine (0.5 mg/kg PO SID), ketoprofen (5 mg/kg PO), intralipid (0.5–1 mL/kg IV SID; INTRALIPID® 20%; Baxter International, Deerfield, IL, United States), pentoxifylline (25 mg/kg PO SID; Pentoxil Upsher-Smith Laboratories, Maple Grove, MN, United States), erythropoietin (150 IU/kg IM; EPOGEN^®^ Amgen, Thousand Oaks, CA 91320, USA), and misoprostol was continued topically. Treatments were performed daily, but the wounds, including significant tail involvement, continued to progress. The animal did not improve, and the vestibular signs persisted. Due to quality of life concerns and poor prognosis, the guitarfish was humanely euthanized.

On necropsy, the most significant finding was the accumulation of tan purulent material and reddening around the endolymphatic pores that followed the contour of the skull and was associated with the inner ear structures. Additional sectioning of the skull showed the accumulation of thick tan material within the labyrinth canals, more severe on the left when compared to the right side. The tail showed signs of chondritis, presumed from trauma during the clinical course. Histopathology confirmed the clinical diagnosis of a bacterial infection within both inner ears (vestibular labyrinthitis). Gram-positive coccobacilli were present in moderate numbers and consistent with the postmortem culture results (pure culture of *Enterococcus faecalis*). A similar milder inflammatory process was evident in the perilymphatic spaces (left more severe than right), consistent with the gross appearance of exudate superficially and cultured the same organism. Both of these findings were the likely cause for the initial clinical signs (circling and disorientation). Serum levels of drugs were assessed to identify if dosages and intervals resulted in levels considered therapeutic in other animals (Table 2). Despite achieving levels in antibiotics that were sensitive to the organism, clinical signs were not abated, suggesting penetration to the target tissues may not have been sufficient.

#### Case 2: Porcupine ray

A 6-year-old female porcupine ray (*Urogymnus asperrimus*) presented after a noted swelling on the midline on the dorsum just caudal to the eyes along with backwards swimming. Fine needle aspiration (FNA) yielded purulent material, and cytology revealed marked heterophilic and histiocytic inflammation with mild eosinophilic and lymphocytic components and reactive fibroplasia, but the animal was otherwise clinically and behaviorally normal and in good body condition. The animal was anesthetized for the examination, and initial treatment consisted of debridement with subsequent wound care and treatments including manuka honey-impregnated gauze (MEDIHONEY Calcium Alginate Dressing; Allegro Enterprises, Bryanston, Johannesburg, South Africa) and amikacin pluronic gel (Amikacin: Polox-A-Gel; Wedgewood Pharmacy, Swedesboro, NJ, United States), along with carprofen (4 mg/kg PO SID; RIMADYL^®^, Zoetis, Parsippany-Troy Hills, NJ, United States), cefpodoxime (5.5 mg/kg PO SID; Simplicef^®^, Zoetis, Parsippany-Troy Hills, NJ, United States), amoxicillin–clavulanic acid (19 mg/kg PO BID; Clavamox^®^, Zoetis, Parsippany-Troy Hills, NJ, United States), and ceftazidime (20 mg/kg IM q72h; Pfizer, New York, NY, United States). *Enterococcus faecalis* was consistently cultured from the abscess throughout the course of treatment. A sand box was introduced to the enclosure to help prevent any wounds from rubbing and resting along the ventral surface.

**Figure 4:**
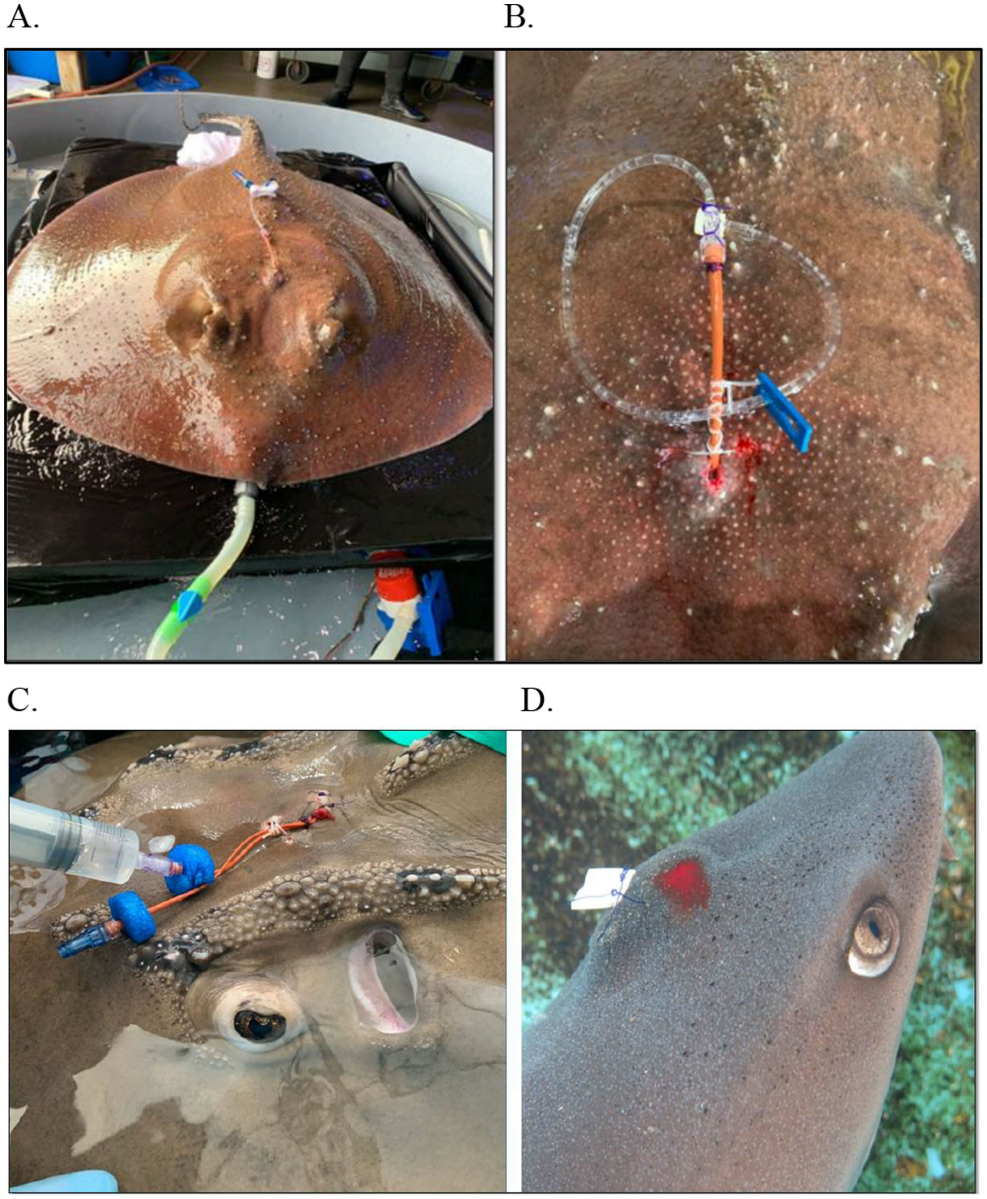
Various drains and debridement as part of a multimodal treatment plan for endolymphatic disease in elasmobranch species. **(A)** Porcupine Ray (*Urogymnus asperrimus*); **(B)** Bowmouth guitarfish (*Rhina ancylostoma*) with flushing; **(C)** White tip reef shark (*Triaenodon obesus*) tagging post aspiration.

**Figure 5:**
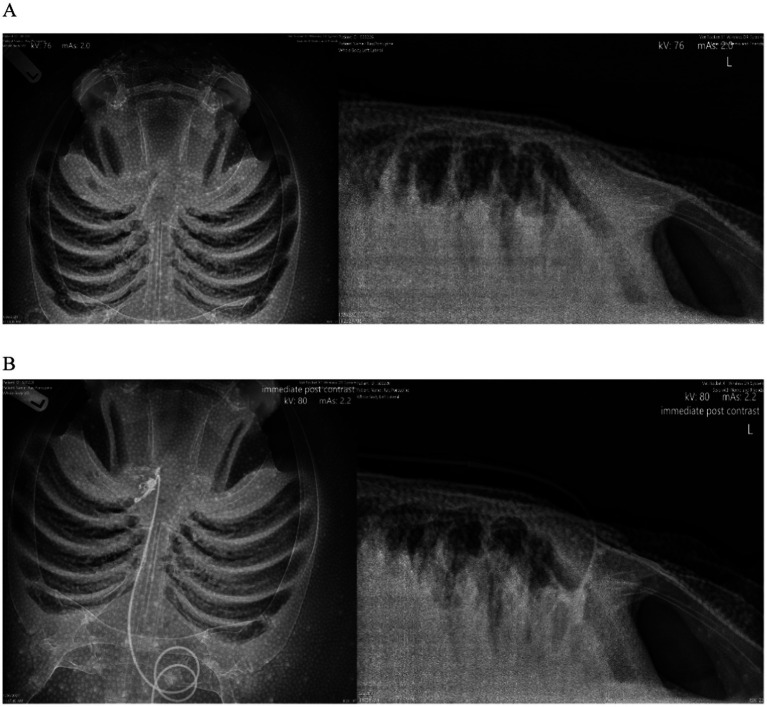
Pre and post contrast radiographic images of a porcupine ray (*Urogymnus asperrimus*) with endolymphatic system disease. **(A)** Survey view, ventrodorsal (VD) view and lateral. **(B)** Post iodinated-contrast infused into the affected area, VD view and lateral.

The swelling improved and the animal continued to remain clinically normal, but the swelling recurred several months later, worse than the initial presentation. On physical examination, the swelling was more pronounced and the original fistulated area was easily re-opened. A more extensive wound exploration indicated association with infection of the ELS, diagnosed by cytology and culture of purulent material aspirated from the area. Debridement and wound flushing proceeded along with the placement of an indwelling catheter through an extant fistula created by disease (Figure 4A). In brief, a red rubber catheter was inserted into the deepest region of the wound (presumably in the perilymphatic spaces) and sutured into place using flexible tubing from an equine subpalpebral lavage (SPL) system (MILA International, Dubai, United Arab Emirates) (Figure 4A). Due to the nature of the skin, a hobby drill was required to make a hole in the skin to thread the tubing and suture an extension set onto the catheter using a Chinese finger trap knot. This pattern allowed a tube to be firmly secured to the skin by a single point, and the physical forces are redistributed along a series of crosses and knots applied around the tube. In addition, the catheter was capped with a one-way valve injection cap (MicroClave^®^ Male Adapter Plug, Manufacturer Item #:10014487; Zoetis, Loveland, CO, United States), and a foam piece was affixed to the end to allow floatation; otherwise, movement in the water resulted in abrasions on the skin with the distal end of the catheter. Radiographs were taken with iohexal (240 mg/mL; GE Healthcare Inc., Marlborough, MA, USA) contrast infused into the catheter to see the extent of the lesion (Figure 5).

Following successful placement, the ray was treated in the water through voluntary training with daily enrofloxacin (Zoetis, Parsippany-Troy Hills, NJ, United States) flushes (mixed with saline at 20 mg/kg) via the catheter for a total flush volume of 60 mL, but this caused erythema and local irritation so enrofloxacin was discontinued but flushes continued with regular saline, which resolved the reaction. The animal was treated with continued parenteral antibiotics and analgesia over time during handlings: amikacin (2.5–5 mg/kg; AMIGLYDE-V®, Zoetis, Parsippany-Troy Hills, NJ, USA), meloxicam (0.1 mg/kg IM; Boehringer Ingelheim, Ingelheim am Rhein, Germany), ketoprofen (2–4 mg/kg IM), danofloxacin (7 mg/kg IM), and florfenicol (40 mg/kg IM; AdvaCare Pharma, 1,623 Central Ave #210, Cheyenne, WY, USA). The ray did well with compliance and treatments with the catheter, and the swelling continued to decrease over time. Parenteral treatments completed but flushes continued for over 2 months at which point the animal was moved back to her exhibit and has had no recurrence of clinical signs to date, several years later. Focal therapy in this case had the most profound and rapid return to normal function.

#### Case 3: Yellow ray

An 8-year-old male yellow ray *(Urobatis jamaicensis)* presented for an acute development of localized swelling along the dorsal midline of the head associated with the endolymphatic pores (31). The animal was otherwise clinically normal, in good body condition, and serial blood work was generally unremarkable. Physical examination revealed a large abscess (confirmed with cytology) with a deep draining tract entering the labyrinth.

Repeated cultures were positive for *Mycobacterium chelonae*. Multiple anesthesia events occurred for debridement and varying wound treatments which included: infusing ciprofloxacin ophthalmic solution followed by insertion of vancomycin pluronic gel (Wedgewood Pharmacy, Swedesboro, NJ, USA); using a small fragment of trimethoprim sulfadimethoxine tablet (approximately 5% of a 960 mg tablet) and placing in the subcutaneous cavity and holding it in place with a small strip of honey-impregnated gauze; packing the tract with ciprofloxacin and clindamycin POP (plaster of Paris) beads, capped with gelfoam and sutured in place with 3–0 PDS into almost complete apposition (Figure 4B); and a mixture of silver collosate and collagen powder (Collasate Silver gel, The Hymed Group, 1817 Apple Tree Lane, Bethlehem, PA, USA). The animal was treated with ceftazidime (20 mg/kg IM q48h), ketoprofen (4–5 mg/kg IM q48h), meclizine (1.5 mg/kg PO SID long term), ciprofloxacin (26 mg/kg PO SID; Pfizer, New York, NY, USA), and danofloxacin (10 mg/kg IM q96hr). The external lesion appeared to be healing well over the course of the treatment period.

Multiple cone beam computed tomography (CBCT) scans with and without contrast were taken over the course of the case. To obtain images with contrast, 2 mL/kg of IV of iohexal was administered via the ventral tail vein followed with 3 mL of elasmobranch ringers IV. The case was resolved after a month of treatment but recurred with a dorsal cranium swelling 1 month later. Abnormal swimming with spinning developed and persisted for the remaining course of treatment. Treatment with meclizine (0.6 mg/kg PO SID) improved the abnormal swimming throughout this period, but the animal’s final examination was 1 year after initial presentation for overall low appetite and continued abnormal swimming. At this examination, due to persistent presence of organism and continued clinical signs, systemic mycobacteriosis was presumed. More intensive treatments were initiated (assist feeding, amikacin (2.5–5 mg/kg IM), azithromycin (10 mg/kg PO SID; Pfizer, New York, NY, United States), and ketoprofen (4–5 mg/kg IM)), and quality of life was discussed. The ray was found dead 3 days later, and necropsy confirmed systemic mycobacteriosis with continued infection in the labyrinth, showing no improvement from treatments; this is likely because focal treatments were limited to the superficial structures of the ELS.

#### Case 4: Bamboo shark

A 9-year-old female brownbanded bamboo shark (*Chiloscyllium plagiosum*) presented with a midline, dorsal head swelling associated with the endolymphatic pores. On initial examination, the area was aspirated and flushed using a 22 g catheter until the fluid ran clean, CBCT was performed, and the animal was treated with ketoprofen 5 mg/kg IM and danofloxacin 10 mg/kg IM, once. Cytology of the lesion revealed necrosis and chronic-active inflammation with reactive fibroplasia. Cultures were negative, and serial blood work was unremarkable. A gram-stained slide from deep samples revealed occasional gram-negative mixed bacteria, including small coccobacilli and diplococci. CBCT survey and an IV iohexol contrast series (as in Case 3) were performed, and there were no abnormal findings noted in the endolymphatic system.

The animal was monitored over the course of 6 months. CBCT with contrast was repeated, and no significant findings were noted. The animal was considered fully resolved with no recrudescence at publication.

#### Case 5: Bowmouth guitarfish

**Figure 6:**
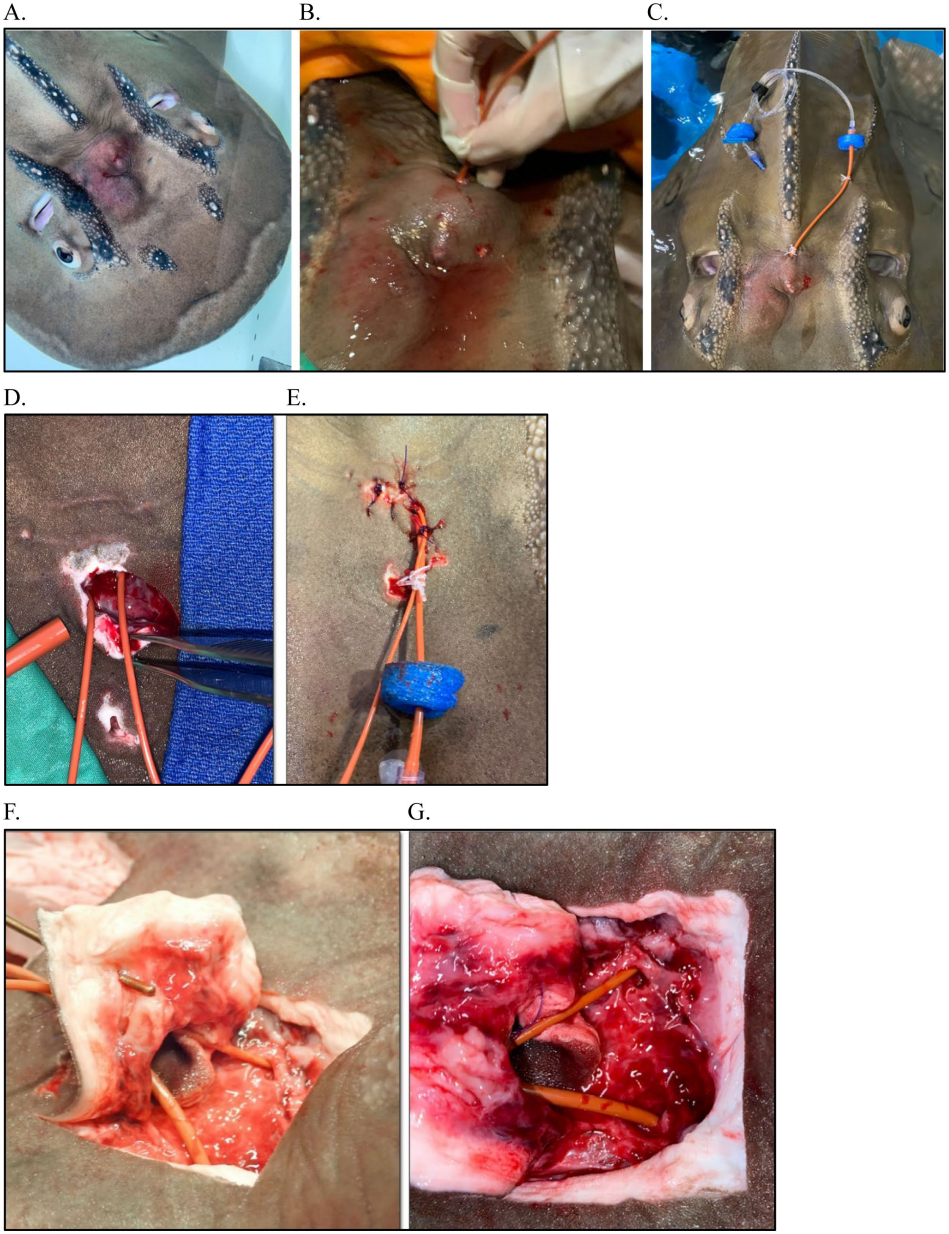
Surgery and catheterization for treatment of endolymphatic disease in a Bowmouth guitarfish (*Rhina ancylostoma*). **(A)** Clinical presentation; **(B)** Flushing the parietal fossa during first exam (Procedure #1); **(C)** Catheterization into parietal fossa during procedure #2; **(D)** Surgical approach to fossa and catheterization into perilymphatic fenestration during procedure #3; **(E)** Closure of surgical site and securing catheter; **(F)** View of catheterization at necropsy with a metal probe inserted into the endolymphatic pore, up close and yellow arrows indicate the endolymphatic pore.

A 6-year-old male bowmouth guitarfish presented for intermittent mild spinning (reported as occasional somersaults). On examination, the animal was found to be in good body condition, and the right endolymphatic pore was erythematous, but with no swelling or discharge while the left pore was normal; no other abnormalities were observed. Additional diagnostics and treatments were not pursued due to the intermittent and improving nature of the abnormal swimming. Six months later, the animal was reported to have a decrease in appetite and marked swelling and erythema of the endolymphatic pore region was noted (Figure 6). Following initial reporting, video and direct observations showed the animal to have an episode where it went onto its back as well as abnormal swimming with notable evidence of vestibular dysfunction. On initial examination, the animal was found to be under conditioned. Blood work showed a leukocytosis compared to in house normal ranges. CSF showed no abnormalities.

Given the previous cases, both systemic and focal treatments were pursued. There were three major treatments throughout the course of disease. For the first, the animal was sedated, and the area of the pores was aseptically prepared. Given the tall dome of the bowmouth, three holes were drilled through the skin (in already thinned areas from presumed pressure necrosis) (Figure 6) to allow flushing and draining: one dorsally and two more ventrally. One liter of Elasmobranch Balanced Salt Solution Salt Solution (Thermo Fisher Scientific, 168 Third Avenue, Waltham, MA United States), 1,000 mL; NaCl, 8 g; urea, 21 g) (32) was flushed through the affected area and then infused with animax ointment (triamcinolone, neomycin, nystatin, and thiostrepton; Animax^®^ Ointment, Dechra Pharmaceuticals, 24 Cheshire Avenue, Northwich, United Kingdom). The animal was assist fed gruel (1% body weight) with 30 mg/kg metronidazole tablets and 6 mg/kg carprofen.

The second procedure occurred the following day with more time to plan a strategy and involved surgical placement of a catheter into the abscess region. In brief, a red rubber catheter, attached to an extension set, was connected with a two nylon finger trap and capped with a one-way syringe adaptor. Two floating foam pieces were attached to maintain the caudal end of the catheter floating to prevent skin abrasion and trauma. The cranial tip of the catheter was inserted through the abscess, directed rostrally into the dorsocaudal hole (made previously at first treatment) and guided through the tissue into the cranial medioventral hole (made using a curved 14 g needle to bore through the skin) and thread with a soft rubber tube from a subpalpebral lavage kit. The catheter was affixed to the caudal area of the abscess (between the pores) with a finger trap and a second securing suture with the same material to the left of the dorsal ridge and a finger trap. The catheter was cut to fit immediately within the abscess but not outside of the cranial hole and drilled holes into the catheter that was within the abscess to provide adequate flushing opportunity. The region was then flushed with elasmobranch ringers infused with 100 mg of ampicillin.

With no improvement 1 week later, the third procedure was performed with two indwelling catheters placed within the inner ears via a surgical approach into the parietal fossa and the area was flushed and antibiotics and steroids were infused. CBCT image reconstruction of the area from case 1 facilitated anatomical understanding. The pores were left intact, an incision was made caudal to the area, and the perilymphatic openings (fenestrations) were visualized as slightly medial to the pores and in-line with the spiracles (Figure 6); the endolymphatic ducts and openings were not seen, and this may be due to the significant inflammation present or anatomical variation. This surgical approach allowed visualization of the sac/fossa area which helped identify the opening to the inner ear and also allow further exploration. An 8 Fr red rubber catheter was placed by approaching from the contralateral side and sliding in from the medial aspect and then curving down and more caudally to get into the inner ear (~7 cm deep) (Figure 6D). This was repeated on the other side. Samples were collected from each side for culture and cytology. Both sides were flushed with 250 mL EBSS, producing copious purulent material from the left side with less material from the right. Flushing continued until the fluid ran clear (~150 mL) at which point the catheters were secured and 5 mL of Solu-Cortef followed by 5 mL ampicillin approximately 15 min later were infused bilaterally. Both catheters were secured in place with 2.0 MonoWeb in the soft tissue on the ventral surface of the sac using a finger trap pattern. Three simple interrupted sutures were placed to oppose the incision, one simple interrupted across the incision, and then a finger trap to keep both catheters together (Figure 6E). An extant stoma was used to thread a 5 Fr silicone tubing from the SPL kit and then finger trap both catheters that were bunched together. Each side was sealed with a one-way syringe valve cap, and small pieces of foam float material were added to the tips to avoid rubbing on the patient’s body (Figure 6E). Flushes were done daily under light sedation.

In addition to the surgical treatments and subsequent flushes/infusions, the following treatments were utilized throughout the course of disease: carprofen 5 mg/kg PO SID, meclizine 0.5 mg/kg PO SID, metronidazole 16 mg/kg PO SID changed to enrofloxacin 10 mg/kg PO SID and ceftazidime 20 mg/kg IV q48hr, intralipid 20% 2 mL/kg IV followed by EBSS 10 mL/kg IV, and misoprostol powder topically to wounds. Drug levels were taken at multiple time points throughout the course of treatment for carprofen, metronidazole, and meclizine which helped guide the course and dosing of treatments.

There were signs of disease progression throughout the course of treatment including increasing frequency and severity of vestibular episodes with some of these episodes associated with tachypnea (respiratory rate > 100 bpm), anemia post-surgery, anorexia, and signs of toxicity on complete blood cell count (CBC) later in the course. However, there was immediate improvement after the third surgery, thus supporting the need for local and aggressive focal treatments.

Cytologic findings were consistent with chronic-active inflammation with chronic hemorrhage, necrosis, and formation of granulation tissue. Some signs of toxicity (foaminess and increased frequency of basophils) on a CBC later in the progression and *Enterococcus faecalis* was cultured from multiple sites including blood and the endolymphatic samples. Vestibular signs improved following the third procedure, but the animal continued to remain anorexic. Despite treatments, the animal was found dead 14 days following initiation of therapy.

**Figure 7:**
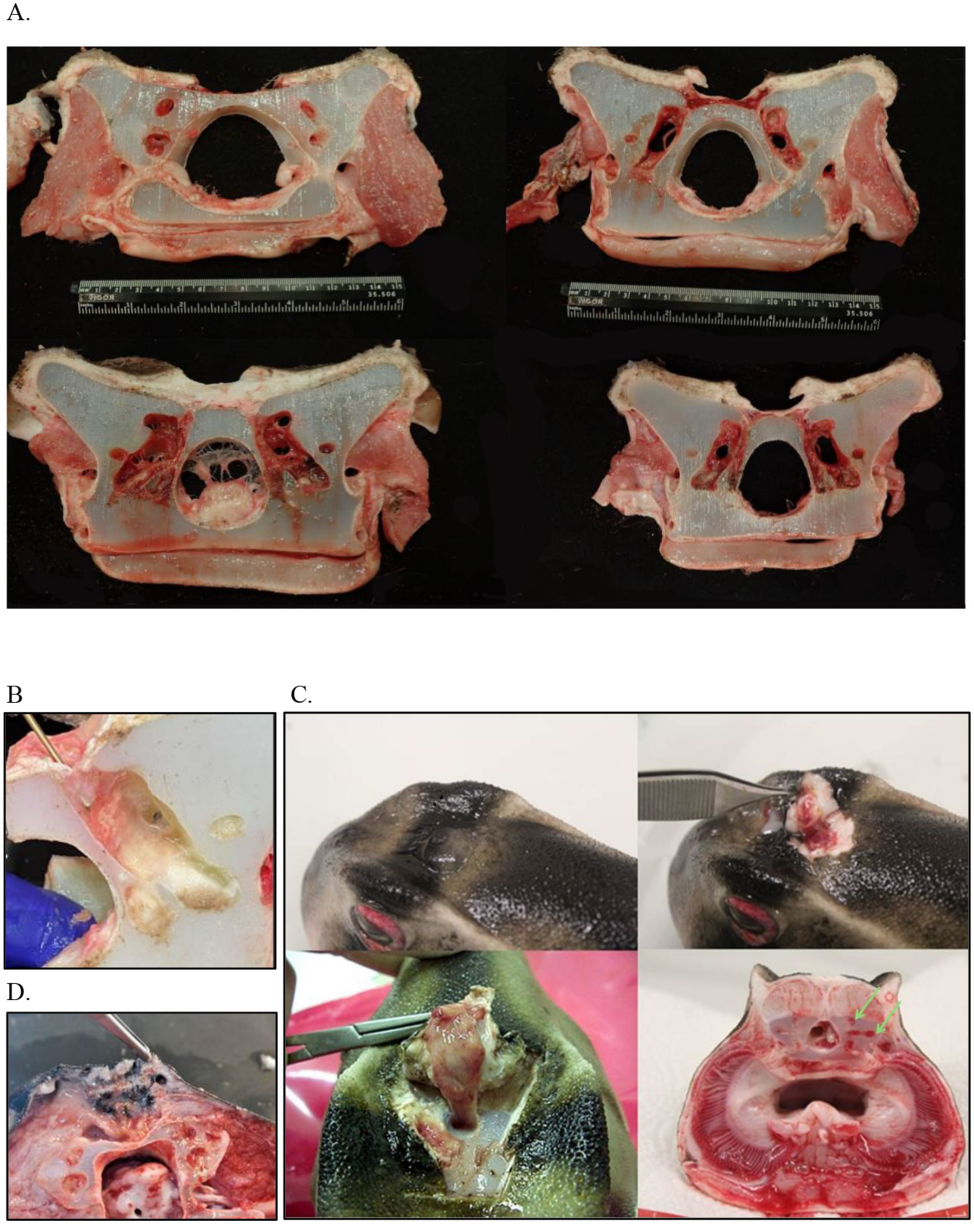
Postmortem examples of endolymphatic disease in elasmobranchs. **(A)** Cranial to caudal cross sections of the inner ear at necropsy in a bowmouth guitarfish (*Rhina ancylostoma*); **(B)** The opening that was catheterized in a bowmouth guitarfish (*Rhina ancylostoma*) #2 from Case Series 1 with the probe is showing the direction of the fenestration into the otic capsule. **(C)** Necropsy of a Port Jackson shark (*Heterodontus portusjacksoni*) with unilateral labyrinthitis. The infected labyrinth (green arrows) shows severe congestion of the semicircular canals. **(D)** Adenocarcinoma in a whitetip reef shark (*Triaenodon obesus*) at necropsy.

The most significant finding at necropsy was the presence of multiple areas of acute necrosis of the gill that were widely scattered throughout the arches as well as moderate infarcts in the liver. This was believed to be the result of sepsis secondary to the ongoing debilitation and the extensive infection within the ELS. Sectioning of the skull and examination of each of the structures of the inner ears and labyrinth systems confirmed the origin of the disease as all these structures were extensively congested and reddened throughout although infection was not overtly evident (Figure 7A). The main conclusion was that this animal had chronic disease of the ELS that likely compromised him in more subtle ways than appreciated.

### Case series 2

Six cases of endolymphatic infections in adult Port Jackson sharks (*Heterodontus portusjacksoni*) were observed over a 22-year period and treated. An original cluster of four cases (1) (three females and one male, all wild born) presented as regional swelling around the endolymphatic pores that were fungal abscesses which also were associated with disease in the EL ducts associated with labyrinthitis and in some animals a non-purulent diffuse meningitis. Ocular lesions such as scleritis, corneal edema, corneal rupture, iritis, and/or exophthalmia were present in three of the four sharks. Histology of the head nodules showed a granulomatous reaction with thin and visible fungal hyphae and heavy cellular infiltration. There were similar fungal hyphae inside the endolymphatic duct cartilage walls. Treatments included enrofloxacin (15 mg/kg PO q48hr for 2 weeks) which was ineffective, but prolonged treatments with ceftazidime (30 mg/kg IM q72hr for 2 months) seemed partially effective in some sharks. Flunixin meglumine (2 mg/kg IM q48-72 hr., three injections total; Finadine^®^; Merck Animal Health, 126 East Lincoln Avenue, Rahway, NJ, USA) was used as an adjunct with some improvement. Itraconazole (20 mg/kg PO q48hr; Sporanox^®^, Janssen Pharmaceutica, 1,125 Trenton-Harbourton Road. Titusville, NJ, USA) administered for 2 months was ineffective in all cases. *Fusarium solani* was isolated from all cultures.

**Figure 8:**
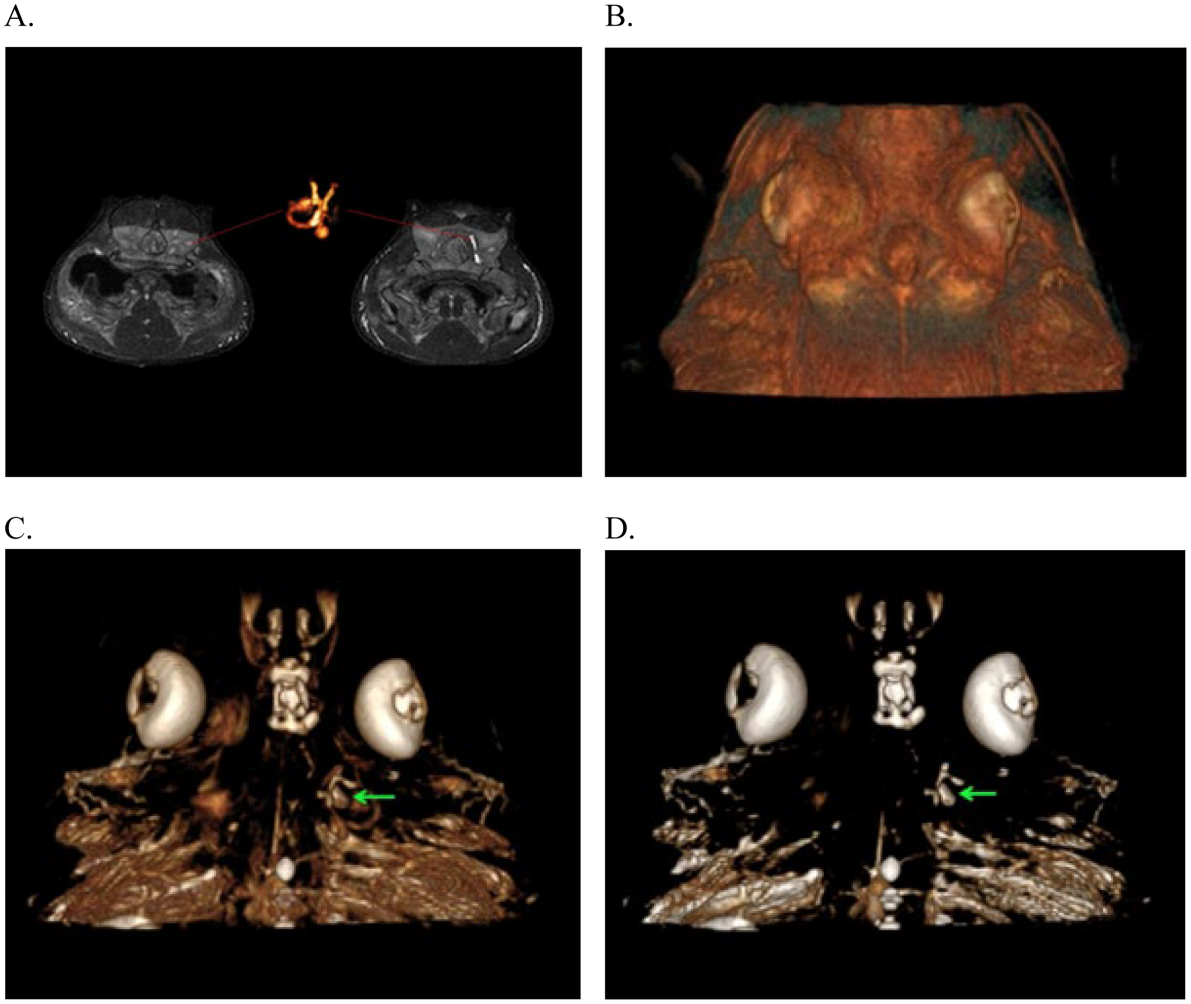
Magnetic Resonance Imaging from the 3D-CISS sequence of the endolymphatic system in Port Jackson sharks (*Heterodontus portusjacksoni*). These images illustrate the absence of signal in the infected left membranous labyrinth. **(A)** Images highlighting the MR signal of the unaffected right membranous labyrinth and its corresponding 3D reconstruction. **(B–D)** 3D reconstruction of the head with successive transparency applied for virtual dissection of tissues to highlight the presence of the membranous labyrinth in the unaffected right inner ear, filled with lymph (green arrows).

Subsequent cases occurred in two males born in the aquarium, 20 years after the first series (2). The first case, a 2-year-old male, presented with a 2-week period of anorexia, and an intermittent circular and spiral swimming pattern to the left side. A dorsal subcutaneous head nodule was visible. Treatment was initiated with terbinafine (20 mg/kg PO SID; Lamisil, Novartis Pharmaceuticals UK, The WestWorks, 195 Wood Ln, London W12 7FQ, United Kingdom) and 10 days later raised to 40 mg/kg and ceftazidime (30 mg/kg IM q72hr). Three weeks later, clinical signs continued with exophthalmia of the left eye manifesting with congested sclera, left gill slit muscle paralysis, and fistulation of the head nodule. Treatments were initiated with meloxicam (5 mg/kg IM q48hr) and focal aspiration of material in the nodule. Clinical signs did not improve, and the animal was humanely euthanized. A postmortem CT scan and magnetic resonance imaging (MRI) were performed (Figure 8).

The CT scan confirmed left-sided exophthalmia with an intact endocranium. MRI allowed greater visualization and contrast of soft tissue structure of eye and the ear showing periocular edema, loss of endolymph in the left labyrinth, and the presence of a fistula connecting the infected parietal fossa with the left eye (Figure 8). MRI was more informative about the inner ear and eye lesions than the CT images, and MRI three-dimensional (3D) constructive interference in steady state (3D-CISS) sequences were more informative than T1 and T2 images.

Histology showed conjunctivitis, labyrinthitis, and meningitis with at least one cranial nerve root affected. There was also mycotic contamination of the labyrinth cartilage. Two bacteria were isolated: *Citrobacter braakii* was cultured from the abscess during the treatment and *Stenotrophomonas maltophilia* from the brain at the necropsy. *Fusarium solani* (complex) was cultured from both areas.

The final case was a 3-year-old male born in the aquarium, who presented with two discrete episodes of circular swimming. With the first episode, examination and ultrasound images of the parietal fossa were normal. The animal was treated for 6 months with ceftazidime (30 mg/kg IM q72hr), vitamin C (30 mg/kg PO SID; Ascor^®^, McGuff Pharmaceuticals Inc., 4,040 W Carriage Drive, Santa Ana, CA, United States), and a combined antifungal therapy of terbinafine (40 mg/kg PO SID) and itraconazole (10 mg/kg PO SID) and assist feeds. Response to treatment was intermittent with no recurrence of the pore swelling. With the second episode, 6 months later, spiral swimming episodes were observed along with swollen endolymphatic pores. The antifungal doses were increased (terbinafine 50 mg/kg and itraconazole 20 mg/kg) but despite 8 months of treatment, the spiral swimming continued to progress, and the shark was humanely euthanized. Blood work was not informative in this case, and *Fusarium solani* was not cultured from this individual. The animal did not have a necropsy because it was reserved for future imaging studies.

### Case series 3

An 8-year-old male lesser devil ray (*Mobula hypostoma*) presented with a change in swimming pattern involving large circles to the right. Blood work showed no changes over baseline for this individual, physical examination, and coelomic and cranial ultrasounds revealed no significant findings; thus, no treatment was initiated as the patient was otherwise eating well and was stable. Three months later, the behavior escalated and the animal began swimming in tighter circles. Empirical treatment was started for a presumed infection and/or inflammation of the ELS with ceftazidime (30 mg/kg IM q72hr) for two doses and methylprednisolone (1 mg/kg) for one dose. With no improvement, amikacin (2.5 mg/kg q72hr) was introduced, which resulted in an improvement of the swimming pattern. The animal returned to normal for 2 weeks followed by recurrence of clinical signs. New blood work showed a significant rise in the granulocytes prompting a change in treatment to enrofloxacin (5 mg/kg PO q48hr), ketoprofen (4 mg/kg IM q48hr), and betahistine dihydrochloride (8 mg/kg q12hr; Betahistine, NHS England London, Wellington House, London). In the following week, there was no improvement with persistent and severe vestibular signs, prompting humane euthanasia. Histopathology confirmed the presence of a granuloma in the right side of the ELS. Bacterial cultures of the ELS, cerebrospinal fluid, and kidney were all negative.

### Case series 4

A 25-year-old male sand tiger shark (*Carcharias taurus*) presented for acute onset of swimming in circles to the right except during feeding sessions. Enrofloxacin (15 mg/kg PO q48hr) was administered for 14 days, and the animal improved after this treatment. Recrudescence occurred a year later showing similar clinical signs with the addition of intermittent anorexia, open mouth breathing with no buccal pumping and flared out branchial slits, and a swollen area around the head’s dorsal midline associated with the endolymphatic pores. Oral enrofloxacin (15 mg/kg PO q48hr) was resumed with the addition of vitamin B complex tablets (3 tablets (each containing 250 mg thiamine, 250 mg pyridoxine, and 500ug cyanocobalamin) PO q48hr; Hidroxil, Almirall, LLC, 101 Lindenwood Dr. #400, Malvern, PA, USA). After a month of treatment, the clinical signs did not improve, and the animal continued to be anorectic. Treatment was then changed to ceftazidime (30 mg/kg IM q72hr), vitamin C (12 mg/kg IM q72hr), and vitamin B12 (2000 μg IM q72hr; Phoenix, Clipper Distributing Company, LLC, 302 S 59th St, St Joseph, MO, USA), methylprednisolone (1 mg/kg IM once; Pfizer, New York, NY, USA), and methenolone enanthate (200 mg IM once; Samex Overseas, Gujarat, India) to stimulate appetite. A FNA from the swelling showed a dense brown material with cytology showing high numbers of red blood cells and some scant white blood cells (WBCs) (mostly heterophils) but no obvious infectious agent was noticed. A CBC identified heterophilic inflammation. When appetite improved, oral treatments were resumed with enrofloxacin, meloxicam (0.2 mg/kg PO q48hr), and terbinafine (10 mg/kg PO SID) based on the suspicion of a fungal etiology affecting the ELS (based on Case Series #2). Despite initial improvement in appetite, the swimming pattern did not change significantly. A second procedure was performed to repeat a FNA, create a stoma, and flush the affected area. Sample cultures did not yield any growth. Oral treatment continued for 3 weeks with partial resolution of the clinical condition. Manageable vestibular signs continued for 14 months after initial presentation until an escalation of clinical signs occurred. Treatments were switched to gabapentin (25 mg/kg PO), and diazepam (0.25 mg/kg PO; Valium®, Roche Laboratories Inc., 9,115 Hague Rd. Indianapolis, IN, USA) was added for appetite stimulation twice per week. The animal did well on this treatment course, and the gabapentin was decreased progressively to 18 mg/kg and diazepam to 0.05 mg/kg. Swimming improved, and the animal had decreased open mouth breathing episodes but approximately 6 months later presented again with tight circle swimming, anorexia, and edema around the pelvic fins and the base of the claspers. Diazepam and gabapentin doses were increased, and vitamin B complex tablets were resumed again (Hidroxil 3 tablets PO q48hr). The animal’s condition improved slightly for approximately 6 months. Over the last week prior to death, clinical signs progressed again, and the animal showed accelerated swimming, active open mouth breathing, anorexia, and small white round skin lesions around the entire body and was found dead prior to the next examination. On necropsy, the region of the endolymphatic pores was swollen, and the parietal fossa and the EL ducts were filled with thickened, brown fluid. In addition, the brain had an asymmetric appearance with a subjective increase in size of the left side as well as branchial necrosis.

A second case presented of a 15-year-old female southern stingray (*Hypanus americanus*) with recurrent episodes of swelling with a fistula on the dorsal area of the head between the eyes, associated with the ELS, and presenting clinical signs were anorexia and lethargy. Initial treatment included with ceftazidime (30 mg/kg IM q72hr) and vitamin B 12 (5 μg/mL IM) and later changed to enrofloxacin (15 mg/kg PO q48hr) and vitamin C (20 mg/kg PO q48hr) for 14 days. The patient improved with the latter treatments. Serial blood work was done, where WBC count (20,000 cells/μl) and creatine kinase (1,185 IU/mL) increased transiently throughout the clinical presentation, but other clinical pathology findings were within normal limits. The swelling on the head was considered fully resolved 13 months post-initial presentation and continues to be quiescent at time of publication. Since resolution occurred, no further diagnostics were pursued.

### Case series 5

A 7-year-old female sandbar shark (*Carcharhinus plumbeus*) developed a swelling along the dorsal chondrocranium centered between the eyes accompanied by poor appetite and abnormal swimming with the mouth slightly open. The swelling continued to enlarge and became erythematous over time. The animal was anesthetized, and purulent material was aspirated from the area for cytology and culture. A 5 mm stoma was made through the skin to allow drainage following flushing with two 60 mL syringes of saline. A heavy growth of *Enterococcus faecalis* was cultured, and the biopsy revealed a lymphocytic and histiocytic dermatitis with fibroplasia and neovascularization. The animal was found dead 17 days after initial treatment. Necropsy showed inflammation around the top of the chondrocranium, while most of the other examined tissues appeared normal. The histopathology report for the brain and spinal cord identified meningitis and mild perivascular lymphoplasmacytic encephalitis. The dorsal skin and vertebra showed regionally extensive cellulitis.

A second 7-year-old female sandbar shark (*Carcharhinus plumbeus*) from the same system was observed with a slight swelling in the same area along the dorsal chondrocranium. The animal was eating well and otherwise clinically normal. Amoxicillin (400 mg PO SID; Zoetis Inc., 10 Sylvan Way Parsippany, NJ USA) was initiated. The swelling resolved, and she remained clinically normal for 3 months. The second sandbar shark was transported to another facility and was initially doing well. The amoxicillin was administered 400 mg with each feeding for just under 3 months. A swelling over the chondrocranium recurred and continued to enlarge. Culture results from the recurrence of the lesion were positive for *Enterococcus faecalis.* The swelling continued to enlarge, and she developed difficulty swimming so she was euthanized about 6 weeks later. Histologic diagnosis of the head lesion was severe inflammation with proliferative, reactive fibroplasia. The brain showed severe histiocytic, neutrophilic, and heterophilic meningoencephalitis.

A third case involved a 4-year-old female white spotted bamboo shark (*Chiloscyllium plagiosum*) that presented anorexic, with an abnormal swimming posture, and a swelling along the dorsal chondrocranium. Treatment was initiated with enrofloxacin (22.7 mg PO) and assist feeding with Hill’s A/D Critical Care soft food (Hill’s Pet Nutrition, Inc., 6,002 N 51st St, Tampa, FL USA). The same procedure and treatments were repeated 5 days later, and an aspirated sample of the area was submitted for culture. A gram stain and cytology showed gram+ cocci, and the antibiotic was changed to amoxicillin–clavulanic acid (125 mg PO SID).

The culture isolated a heavy growth of *Enterococcus faecalis,* and a second culture was repeated 3 weeks later which showed repeated results. Blood culture was negative for aerobic and anaerobic organisms. After 2 months of daily treatment, the shark began eating on her own and was clinically normal. She remained clinically normal for 6 months when signs recurred, and she progressively deteriorated over a week and was euthanized. On gross necropsy, there was inflammation and fluid causing swelling along the dorsal chondrocranium.

### Case series 6

Six cases of ELS disease were identified and treated in whitetip reef sharks (*Triaenodon obesus*). All cases presented over a 5-year period in juvenile to sub-adult sharks (1–4 years of age) including five females and one male. The most common presenting signs were focal swelling of the region surrounding the endolymphatic pores, anorexia, and vestibular signs including abnormal swimming patterns, barrel rolling, head tilting, and resting on the bottom in lateral or dorsal recumbency. One case presented with a “scoliosis-like” abnormal body posture, erratic swimming with head tilt, and anorexia and often would be observed laying in lateral or dorsal recumbency when at rest.

Recurrence of clinical signs was observed in 5/6 sharks at intervals of months to years between events. Serial cultures were obtained at initial presentation and during episodes of recurrence in four of the six sharks. *Enterococcus faecalis* was cultured from all the abscesses with pure growth of *E. faecalis* reported in 9/11 samples. One case also cultured positive for *Staphylococcus epidermidis*-methicillin resistance. The high frequency of recurrence was likely due to recrudescence or persistence of deeper infection.

Based on the culture sensitivities, amoxicillin (80–100 mg/kg PO SID; US Antibiotics, 201 Industrial Dr., Bristol, TN, USA) was started. Other treatments included enrofloxacin (10 mg/kg PO SID), ciprofloxacin (19 mg/kg PO SID), danofloxacin (10 mg/kg IM q5days), carprofen (3.3 mg/kg PO SID q5days), meloxicam (0.2 mg/kg IM or PO q24-48 hr), metoclopramide (0.5 mg/kg IM or PO q24-48 hr), and/or vitamin C (100 mg/kg PO SID). Ceftiofur (6.6 mg/kg q7days; EXCEDE^®^, Zoetis, Parsippany-Troy Hills, NJ, United States) was used in two of the initial cases, but it proved ineffective with no change in clinical signs. Antibiotic treatment was switched from injectable to oral administration when the sharks began to eat. With instances of recurrence, oral amoxicillin was used concurrently with danofloxacin injections. Antibiotic treatment typically continued for 2–4 weeks past the cessation of clinical signs (resolution of pore swelling). NSAIDs were used in half of the cases, and their use was positively correlated with the return of normal food consumption. Two of the cases received 1–5 oral or injectable doses on either meloxicam or carprofen on consecutive days. The third case received meloxicam every other day for 34 days. Two sharks with recurrent lesions were maintained on long-term vitamin C therapy at half of their initial treatment dose. Additional treatments were used in certain cases depending on the clinical signs and condition of the animal. Cold laser treatment was used (EVO MLS, multiwave locked system, VET Therapy Laser machine-Class IV laser) at 6.44 Joule and 2.05 J/cm^2^ for 9 s applied to the affected area. Clinical improvement was observed with lancing of the abscess accompanied with flushing and debridement. In one shark, the abscess was opened with a 2.5 cm incision and flushed with saline and amikacin. A Penrose drain was sutured in place to prevent immediate closure of the wound. The drain was removed 4 days later, and the wound closed by second intention. No significant difference was noted between simple lancing and flushing *vs* short-term drain placement in any of the cases.

In 4/6 cases, the sharks developed sub-optimal body condition over time, in spite of maintaining normal dietary intake and being otherwise asymptomatic for months to years. Five of the six affected sharks died, but mortality was not immediately associated with ELS disease in four of the cases. In these four cases, there was no evidence of active disease for months to years prior to death, so the ELS region was not examined during necropsy.

In the fifth case, the animal had a mild swelling associated with the endolymphatic pores for more than 5 years but had no other clinical signs. In addition, there was no change noted in the swelling following a course of antibiotics administered for a different problem. At 8 years of age, he presented with recurrent bouts of intermittent anorexia. Nine months later, it was observed that the swelling on the head was significantly larger than previously and had developed a cobblestone appearance. Localized soft swellings were identified via palpation and ultrasound; purulent material was observed seeping from these sites. Culture revealed a pure growth of *Enterococcus faecalis*. After 1 month of waxing and waning appetite, metoclopramide (0.5 mg/kg q48hr) was added to the meloxicam (0.2 mg/kg q48hr) and amoxicillin (90 mg/kg PO SID) treatment protocol for this shark. This addition appeared to help stabilize the animal’s appetite in the weeks prior to death. On necropsy, transection through the swelling on the head revealed a nodular mass that was a mottled gray and white color dorsally (1 cm) and medium yellow color ventrally (1 cm) (Figure 7D). The mass extended from the skin down to the chondrocranium where it caused areas of thinning of the cartilage. There was multi-focal abscessation present bilaterally along the periphery of the mass. On histopathology, the mass was identified as an adenocarcinoma that had metastasized to the heart. This shark was also reported to have regionally extensive, marked lymphocytic meningitis. It is hypothesized that the chronic inflammation in this area may have promoted the development of this neoplasm as has been described in other species (33, 34).

The sixth case continues to show cyclical disease and is treated routinely at the time of publication.

## Discussion

Endolymphatic system disease involves the endolymphatic pores, parietal fossa, endolymphatic ducts, and the inner ear of elasmobranchs. It has variable clinical presentations, with the two most common being a swelling on the dorsal chondrocranium and abnormal swimming patterns. Swimming characteristics include spiraling or rolling and repeated directional circling that frequently corresponded with location of disease. These clinical signs are shared with a variety of diseases including central nervous system inflammation or infection [meningitis or encephalitis (e.g., scuticociliates)], skeletal abnormalities [wing ray (ceratotrichia trauma), vertebral malformations or misalignments, and pelvic fin conformational abnormalities], eye lesions, biotoxins, and, in some cases, stereotypy. Disease often waxes and wanes with chronicity often ranging from months to years. It is underrepresented as a differential in neuropathic disease and is difficult to diagnose and treat.

Cases in this series were divided into generalized categories: (1) superficial disease (involving the pores, fossa, ducts, and possible dorsal chondrocranium) and (2) complex disease (involving the deeper structures—perilymphatic and endolymphatic spaces and labyrinth). The latter category always involved neurologic signs. Usually, complex disease was accompanied with superficial signs. Some cases did involve ocular structures and components of the central nervous system which are highlighted separately.

Table 1 presents a summary and breakdown of all cases discussed herein. The anatomically affected areas were varied with (8/23) manifesting superficially as a swelling along the dorsal chondrocranium with no apparent inner ear involvement. 15/23 animals presented with some form of neurologic signs. Many of these individuals had swelling along the dorsal chondrocranium as well but have been classified as ‘complex’ due to the presence of neurologic clinical signs at presentation. While providing a framework for staging this disease process is ideal, it was not possible with this data set due to the great variability among the cases. The authors attempted to start this process by the categories of ‘simple’ versus ‘complex’. However, true staging of this disease process would be ideal and warrants future consideration and investigation.

21/23 animals had cultures performed as part of the diagnostic workup (Table 1). Of the 21 cultures, one revealed no growth, 12 isolated *Enterococcus faecalis*, six identified *Fusarium solani*, one isolated *Mycobacterium chelonae*, and two had mixed organisms. 19/23 of the affected animals died with 5/19 euthanized and the others dying naturally. 14/23 animals had the endolymphatic system examined on necropsy. 13/23 animals had recurrence of disease with 4/13 recurring multiple times. Only 4/23 animals are currently alive and resolved from all endolymphatic-associated clinical signs.

Blood work was generally not helpful with diagnosis in these cases, and any alterations were secondary to consequences of the disease (trauma to muscle, anorexia/loss of body condition, and secondary infections). Some general trends included increased total white blood cell counts above normal, increased granulocytes in affected animals, signs of toxicity (35) among white blood cells, anemia, and increases in muscle enzymes later in disease progression. In elasmobranchs, low numbers of neutrophils in addition to more frequent fine eosinophilic granulocytes (heterophil equivalent), coarse eosinophilic granulocytes (eosinophil equivalent), and basophils are observed (36). Left-shifted neutrophil and heterophil populations have been observed in the blood of various elasmobranch species, while abnormal granulation tends to be less prominent in elasmobranchs than in other non-mammalian vertebrate taxa (35). Though blood work was generally not helpful clinically, it is recommended as a minimally invasive diagnostic and may help with long-term management and tracking of trends over time.

**Figure 9:**
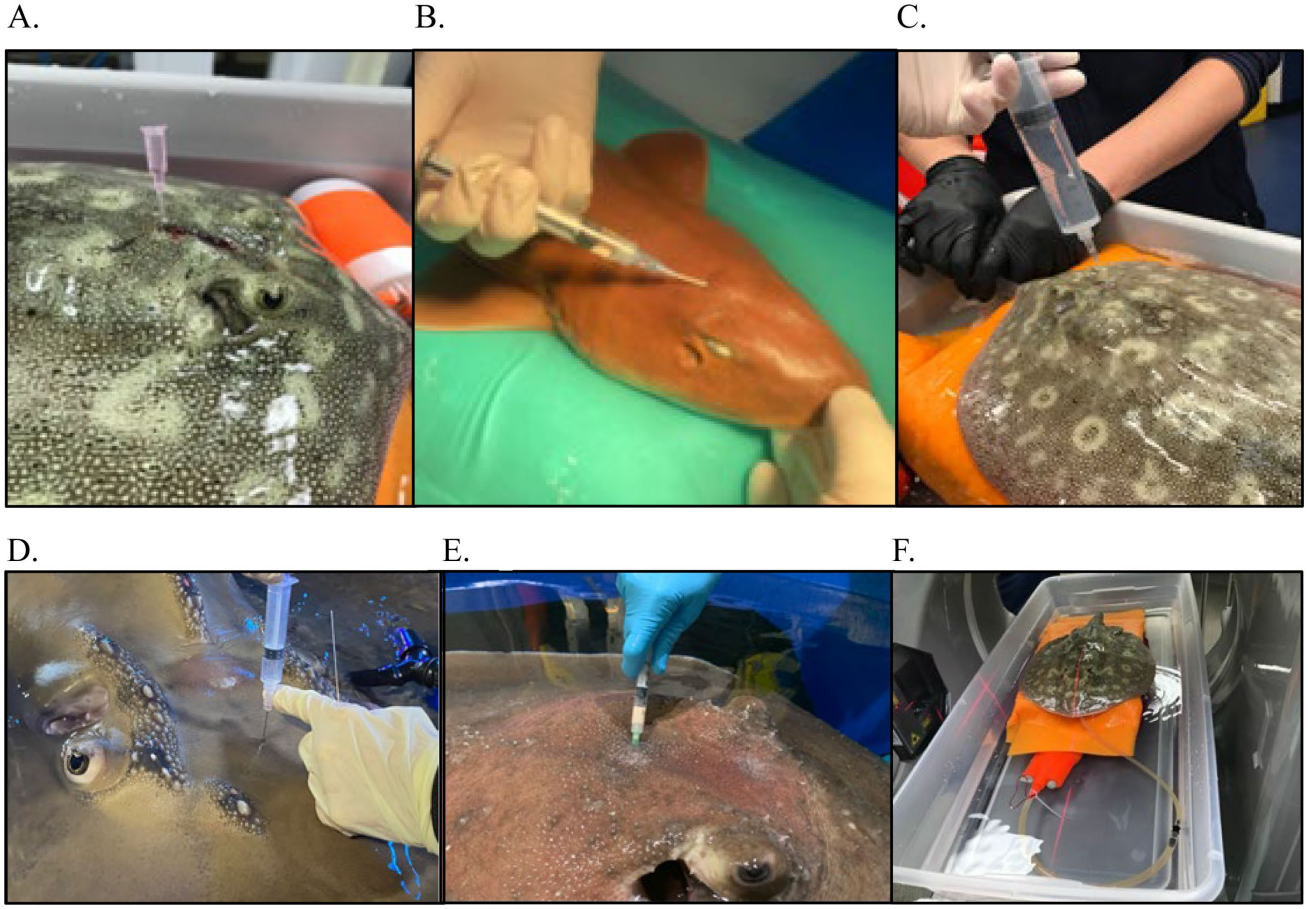
Examples of various diagnostics for endolymphatic disease in elasmobranchs. **(A)** Marking of affected area for sample collection and treatment administration in a yellow ray (*Urobatis jamaicensis*); **(B)** Flushing of affected region as part of debridement in a white-spotted bamboo shark (*Chiloscyllium plagiosum*); **(C)** Flushing the affected region as part of debridement in a yellow ray (*Urobatis jamaicensis*); **(D)** Abscess aspiration in a porcupine ray (*Urogymnus asperrimus*); **(E)** Cerebrospinal fluid (CSF) collection in a bowmouth guitarfish (*Rhina ancylostoma*); **(F)** Cone-beam computed tomography of an anesthetized yellow ray (*Urobatis jamaicensis*), ventilated while raised out of water to maximize image quality.

It can be challenging to obtain diagnostically significant samples due to unique anatomy (small endolymphatic pores and difficult to reach areas of the labyrinth), an aquatic environment, and equipment or clinical limitations. Anatomically, the pores and duct openings are different among species and hard to access. It is critical to understand species differences to facilitate successful sample collection. Aspiration of material from superficial structures is straightforward, but deeper sampling required catheterization or with advanced disease resultant fistulas provided access to deeper affected tissues. Cerebrospinal fluid (CSF) evaluation was used to rule out meningitis or encephalitis in Case Series 1 (Figure 9). Cultures (aerobic, anaerobic, mycobacterial, fungal, and PCR for scuticociliates) and cytology from aspirated material proved to be useful in guiding treatment.

In one series, all necropsied cases of Port Jackson sharks (5 of the 6 cases) found *Fusarium solani*. *F. solani* infections in elasmobranchs have been described mainly with skin, lateral line, skeletal muscle, and cartilage (37–40, 72). They are environmental saprophytic fungi and are important causes of human and animal infections due to the production of mycotoxins and are often associated with temperature related stress (7). Previous literature refers to chondritis lesions in other elasmobranch species, although in those cases it was found in juvenile animals (39, 41–43, 72). *Enterococcus faecalis* was cultured from the blood and/or endolymphatic system samples in 12/23 of the current cases. Delaune and Anderson (44) reported culturing *Enterococcus faecalis* from a spotted eagle ray (*Aetobatus narinari*) that presented due to swelling located on the dorsal head originating from an abscess in the brain. The animal was treated and made a full recovery while never exhibiting abnormal neurologic signs (44). Enterobacteriaceae have been found as intestinal inhabitants of terrestrial and marine vertebrate species and may be normal flora but have also been considered opportunistic pathogens (45, 46). *Mycobacterium chelonae* was also cultured in the yellow stingray case, and this organism has been found as the causative agent of disease in other species, including guitarfish (47). Chang and Okihiro (5) found that the most common cause for meningoencephalitis was bacterial or protozoal with the most commonly isolated agent being *Carnobacterium maltaromaticum*. A study looking at blood cultures from healthy captive and free ranging stingrays found that a single positive blood culture without other corroborating diagnostics (e.g., gross lesions or histologic confirmation) is not sufficient to confirm septicemia in elasmobranchs (48). Repeatability of tests, consistency of results from different sources in the body, and white cells engulfing morphologically appropriate organisms over time may increase the validity that certain organisms are normal microflora *vs* pathogenic.

**Figure 10:**
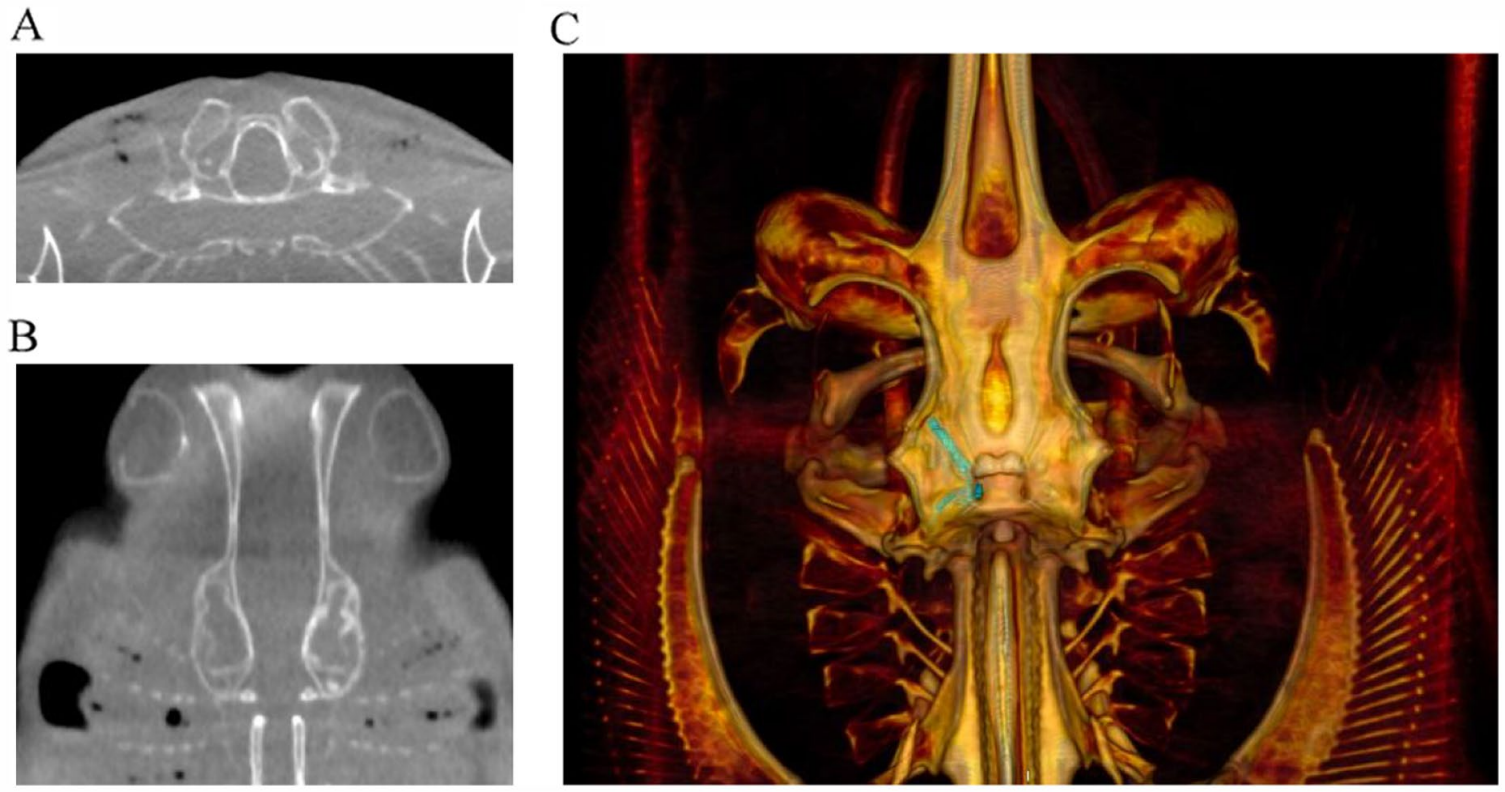
Examples of cone beam CT (CBCT) images of right-sided otitis media in a bowmouth guitarfish (*Rhina ancylostoma*). **(A)** Transverse plane and **(B)** dorsal plane image demonstrating the expansile appearance and thinning of the mineral margins of the otic capsule. **(C)** Volume rendered image of the same individual viewed from dorsal with the endolymphatic duct segmented in green.

Imaging can be difficult in elasmobranchs depending on the targeted anatomical area. Ultrasound would be ideal but of limited either by dense dermal denticles in several species or due to the anatomical location, size of the ducts, and depth of the labyrinth encased within cartilage, all of which are not physically possible to scan. Ultrasound could be useful, where skin texture allows, for clinical evaluation of swelling on the dorsal chondrocranium but the area below is not penetrable by ultrasound waves. Radiographs have some value but can be technically difficult to obtain, and the images acquired may not allow a detailed enough view of the severity of disease (Figure 5). The best modality is computed tomography (CT) or magnetic resonance imaging (MRI). MRI offers superior contrast for the examination of soft tissues when compared to CT. MRI images can significantly enhance characterization of lesions within the inner ear and eyes. Notably, the images (Figure 10) obtained through the 3D-CISS sequence exhibited heightened efficacy in lesion discrimination compared to T1-weighted and T2-weighted images. Fluid attenuated inversion recovery (FLAIR) is an additional sequence that nulls/reduces signal of fluids with low protein and cellularity, such as cerebrospinal fluid (CSF) and endolymph. When the composition of the endolymph is altered, FLAIR sequences can declare this by an abnormally persistent high signal. Though superior for images, MRI is technically more challenging to accomplish. CT is more readily accessible, faster, and excellent for imaging the musculoskeletal system, including the otic capsule. CT’s value in diagnoses is demonstrated in the yellow ray in Case Series 1 and how it helped guide the trajectory for sampling or catheterization.

Treatment approaches among the cases were highly varied, and though many of the treatment modalities did not appear to cause harm, it is challenging to decipher what may have directly resulted in positive outcomes. Several strategies resulted in remission, but recrudescence of clinical signs occurred in more than half of the cases. This is likely because disease did not truly resolve but became quiescent with waxing and waning clinical signs. Complete resolution occurred with simple and superficial disease. Complicated disease with deep infection was difficult to treat and was associated with chronicity. Direct treatments in the region, while difficult, showed clinically relevant changes in microbial loads, inflammation, and clinical signs. Catheterization into the pore and then the ducts was not feasible in these cases, even in the largest elasmobranchs, due to the size of the pores and the inability to see the orientation of the ducts, a ‘natural path’ was not identifiable or palpable. A surgical approach to catheterize into the otic capsule and the perilymphatic or endolymphatic spaces may be necessary to effectively reach the area for either a sample or direct flushing and treatments. As there were not enough cases to determine whether there were long-term negative consequences, there should be a consideration for damage to the fenestral membrane and other otic structures. Given the degree of disease seen and the variability of anatomy, it is unclear if catheterization is going into perilymphatic space or directly into the labyrinth. However, deep exploration has identified purulent material in these areas with significant tissue inflammation and trauma that has already occurred. In at least Case 5, series 1, the fenestral membrane was not appreciated in either foramen and the ducts were also not seen. It was unclear if the latter was anatomical variation or if disease resulted in enough tissue damage as to destroy extant ductal tissue. Negative outcomes in these cases were associated with chronicity and secondary consequences of persistent inflammation, anorexia, and neurologic signs. Euthanasia was elected in five cases, either due to worsening or an inability to improve the welfare of the patient. When death occurred naturally, it was not always anticipated.

Catheterization of the perilymphatic space was successfully performed in the second bowmouth guitarfish in Case Series 1 (Figure 6). Despite being an invasive surgical procedure, it resulted in resolution of the neurologic signs as soon as the animal recovered. It was determined that the endolymphatic ducts could not be catheterized via an approach through the pores, in this species. The anatomy is very intricate, and the curve was too extreme for an approach through the pores that would ultimately damage the endolymphatic ducts. Rather, the approach needs to be made via surgically created access to the chondrocranium, avoiding the endolymphatic pores (Figure 6). This procedure was successful in the resolution of neurologic signs of the animal, but the animal likely already septic and refractory to treatments and ultimately died. If this procedure had been implemented sooner, the outcome may have been different. Certainly, focused and direct treatments are recommended and warrant further exploration.

Several supportive care and ancillary treatments had positive effects, while others had equivocal effects. Meclizine was used with improvement of spinning behaviors in multiple cases, and one case had evidence-based data showing its clinical effects (31). Assist feeding was required in several cases due to chronicity of disease resulting in hyporexia and anorexia; this maintained those patients throughout the course of disease. The addition of a sandpit or sandbox to the holding area was helpful at minimizing wounds and other abrasions that could have resulted from the animal resting on the bottom. Antibiotic-impregnated beads were used in several cases with varying levels of success. Sterilized plaster of Paris (POP), hydroxyapatite, and Matrix III have all been studied and are bioabsorbable beads. POP is inexpensive, and there are various studies looking at the elution properties of various antibiotics from POP beads (49, 50). There were often challenges with application of drains due to the nature of elasmobranch skin but elements of the subpalpebral lavage system were the most useful in Case Series 1 and facilitated focal treatment and regular flushing (Figure 4). Some treatments such as laser therapy and betahistine have only been rarely described in veterinary literature and in the case of betahistine, only in dogs and cats (51–53); it is unclear in those cases how effective these treatments were.

The ability to provide focal topical treatment (most notably flushing) in the affected area made a significant difference in several cases. In cases with superficial infections, resolution occurred with minimal treatments and shorter courses. For the more severe cases in Case Series 1, the bowmouth guitarfish and porcupine ray had catheters placed to allow focal treatment. The previous cases showed sufficient evidence of refractory disease with treatments that theoretically should have been effective. The authors appreciated a significant impact from this focal approach with the clinical course and improvement of disease. Though more invasive, the effect was greater than systemic treatments. In contrast, presumed therapeutic levels of metronidazole did not abate *E. faecalis* infection that was sensitive to the antibiotic or improve disease. It was posited that antibiotics did not reach the tissues parenterally. Early treatment is likely to have a better outcome.

A thought experiment for further treatment included consideration for ablation of the inner ear tissue mechanically via the fenestration via laser, hydrothermal, or possible chemical ablation (54–56). In addition, some postmortem investigations were conducted to assess the feasibility of a traditional modified ‘bulla osteotomy’ and total ear canal ablation. Depending on the species, this may require significant traversing of thick cartilage to get to a very small and narrow space. This would likely require advanced imaging, such as fluoroscopy, for guidance into the correct areas. The spiracles in rays may provide a site of entry.

Lack of pharmacokinetic and pharmacodynamic data is a deficit in elasmobranch clinical medicine. In lieu of that, evaluating drug levels was very helpful in Case Series 1 to help guide treatments and dosing. For instance, some medications had no or low levels at the intended dosing and interval, such as ketoprofen, thus suggesting this plan was ineffective. Meclizine dosed at 1.5 mg/kg PO SID produced levels at 2900 ng/mL which was considered presumably higher than desired (57). While not overtly causing problems, in an abundance of caution, this prompted a change in the dosing regimen. In one of the cases using metronidazole, additional neurologic signs manifested, and serum levels had accumulated to higher than expected values and once removed, signs improved. Carprofen was used in Case Series 1 and showed levels that are therapeutic in other species (Table 2). There is a lot of variability in the pharmacokinetics of drugs, especially between different species.

A combination of multiple diagnostics including serial blood work, culture, cytology, ultrasound where feasible, advanced imaging, and CSF collection all helped with diagnosing the disease and managing treatment. Postmortem examinations were crucial to characterizing disease and for elucidating anatomy for future cases. In reviewing available literature (4, 40, 58), information on endolymphatic disease is minimal with only one case highlighting otitis interna in a white tip reef shark (58), although other cases are alluded to. Since ELS disease is likely underdiagnosed antemortem, it is also highly likely it is missed postmortem as the inner ear is not typically included in a standard necropsy tissue collection set. The importance of always examining the inner ears during elasmobranch necropsies cannot be under emphasized in neurologic cases, especially as described in this study. One reference suggested that the occurrence of disease is more frequent in small sharks (58), but the present case series expands that notion with increased variety of species and size of patient. That reference also adds an additional differential of trauma (usually pore and sac) resulting in secondary infections, which was not identified in this case series. In addition, bilateral disease is referenced, which was not always easy to determine or reported in the cases presented here.

Etiology of this condition is unknown but is most likely multifactorial. The primary pathway for pathogens to enter the ELS would likely be through the endolymphatic pores into the endolymphatic ducts. For most elasmobranchs, the presence of pathogens in the water poses the primary threat for colonization or contamination of the ELS via these pores. Among all the cases, bacterial presence was common, and second most frequent were fungi. It is possible in those cases they were primary pathogens, but secondary presence is possible as a complication of chronicity. In Case Series 2 (Port Jackson sharks), the primary pathogen was considered to be *Fusarium solani*. Poor culture recovery of microorganisms can also be a factor in lack of diagnosis as aquatic organisms require certain media and temperatures, as well as sample handling parameters that promote good recovery of organisms.

One etiologic consideration is an abnormal composition or absence of otoconia in the labyrinth of animals under managed care. An absence of otoconia may affect hearing less than vestibular functions such as posture, balance, and perception of gravitational forces. In elasmobranchs, hearing relies on two mechanisms: a non-otolithic one that perceives sound through the parietal fossa and the macula neglecta (which lacks otoconia), while the other mechanism detects sound through the otoconia or otoconial mass present in the sacculus (6, 16). Vestibular functions are associated with otoconial mass-associated organs—the utricle, saccule, and lagena (6, 16). Further research is necessary to understand the development of exogenous otoconia in the inner ear elasmobranchs with exogenous otoconia under human care and if this is a factor in developing ELS disease. In addition, another consideration could be through the normal formation of exogenous otoconia, such as sand particles, which traverse the ELS into the deep labyrinth (25, 59). In several elasmobranchs, it is assumed that there is a mixture of exogenous and endogenous otoconia, except for Port Jackson sharks, which only have exogenous otoconia, potentially rendering them even more susceptible to ELS disease. This may be one of the reasons why such pronounced fungal infections were found only in Port Jackson sharks in these clinical cases. This potential process may impact the management of this species under human care as Port Jackson sharks born in human care facilities may entirely lack otoconia due to lack of tank substrate or inappropriate material. It is unclear if this is a factor in other species. Further research is necessary to clarify any potential problems in the inner ear development of elasmobranchs with exclusively exogenous otoconia born in human care systems and whether this is a factor in developing ELS disease.

There are many challenges associated with ELS disease, and one of the major ones is prevention or early detection due to the delayed presentation of clinical signs, chronicity, and complex nature of the disease. Thus, the authors advocate for routine preventative medicine examinations to include careful examination of the endolymphatic pores. In addition, aquarium staff should conduct focused observations of the EL pores as well as recognize clinical signs for prompt evaluation. Consideration for advanced imaging (especially with recurrence), while difficult, will help with diagnosis and extent of disease and provide data for species-specific anatomy. With recrudescent disease, a thorough evaluation is necessary to identify if more diagnostics or intensive treatments are warranted. If there are neurologic clinical signs, this indicates inner ear involvement which should prompt a higher level of diagnostic screening.

The majority of cases in this manuscript presented with swelling on the head or spinning behavior. The histories frequently showed chronicity, waxing and waning signs, and recrudescence of clinical signs were common. There were secondary complications including epithelial and ocular lesions (conjunctival edema, hyperemia, corneal edema, iritis, and exophthalmia), inflammation of the endolymphatic ducts, damage to the inner ear, and opportunistic infections from damage to skin, fins, and tails in animals that do not routinely rest or come in contact with environmental elements. In one Port Jackson shark, unilateral paralysis of the gill slits was observed, which was attributed to cranial nerve root injury identified histologically. The severity of disease can be variable and is related to the unique anatomy of the ELS, which will vary based on species. It is unclear why the ELS may be more prone to microbial infection, but a possible explanation may be the increased chance of pathogen exposure at these areas and the role of biofilms and microbiome. Recent literature suggests that the microbiome of wild *vs* managed care animals is markedly different (60, 61). Given the high proportion of *Enterobacter* identified, this should be a consideration. Causative factors and mitigation strategies are theoretical and beyond the scope of this document.

Understanding this unique anatomy is crucial for diagnosis and then developing and carrying out treatment plans. If disease was superficial, there was often a good outcome when it was identified early and treatment was initiated. If the infection was deeper or more extensive involving the inner labyrinth, then intense focal therapy was likely indicated. However, gaining access to these locations is not simple due to the convoluted and intricate nature of the anatomy. Advanced imaging was helpful for many of these cases for monitoring progression and gauging prognosis.

## Conclusion

Endolymphatic disease encompasses a wide array of pathologies that are capable of affecting elasmobranchs. Understanding the anatomy of the elasmobranch ELS and the differences between the species will help to better understand the etiologies and clinical signs of disease. Chronic or recurrent disease can be occult and very challenging to treat. The unique and intricate anatomy can make treatments challenging and penetration to target tissues questionable. Focused treatments seem essential to avoid recurrences of disease. It is recommended to routinely check the endolymphatic pores during routine examinations of elasmobranchs. Advanced imaging can help with guiding treatment plans and gauging prognosis. Most importantly, always include the inner ear tissues in elasmobranch necropsies. Endolymphatic disease appears to be an underdiagnosed condition among elasmobranch species, and more awareness and knowledge on the specific anatomy, physiology, and pathology may prompt timely diagnosis and treatment.

## Data Availability

The datasets presented in this article are not readily available in accordance with privacy protection guidelines. Requests to access the datasets should be directed to the corresponding author.
